# Steatotic liver disease trajectory: A holistic EASL guidance on the multidisciplinary approach to screening and patient management

**DOI:** 10.1016/j.jhepr.2026.101798

**Published:** 2026-02-25

**Authors:** Willem Pieter Brouwer, Luisa Vonghia, Maja Thiele, Jörn Schattenberg, Foteini Anastasiou, Sonja Beckmann, Elisabetta Bugianesi, Laurent Castera, Cyrielle Caussy, Ahmed Cordie, Ruth De Bruyne, Núria Fabrellas, Gema Frühbeck, Zouhir Gadi, Amalia Gastaldelli, Anja Geerts, Laura Haigh, Kate Hallsworth, Adriaan Holleboom, Carolin Lackner, Jeffrey V. Lazarus, Juan Mendive, Maurice Michel, Vicki Mooney, Vlad Ratziu, Michael Roden, Arun J. Sanyal, Wendy C. Spearman, Horia Stefanescu, Dina Tiniakos, Emmanuel Tsochatsis, José Willemse, Faiez Zannad, Shira Zelber-Sagi, Aleksander Krag, Sven Francque

**Affiliations:** 1 Erasmus MC University Medical Center, Rotterdam, The Netherlands; 2 Department of Gastroenterology and Hepatology, Antwerp University Hospital, Antwerp, Belgium; 3 InflaMed Centre of Excellence, Laboratory of Experimental Medicine and Paediatrics, University of Antwerp, Antwerp, Belgium; 4 Center for Liver Research, Odense University Hospital and University of Southern Denmark, Odense, Denmark; 5 Department of Internal Medicine II, Saarland University Medical Center, Homburg, Germany; 6 4rth TOMY –Municipality & Academic Practice of Heraklion Crete, Greece, European Society for Primary Care Gastroenterology, Greece; 7 Center for Clinical Nursing Science & Department of Gastroenterology and Hepatology, University Hospital Zurich, Switzerland; 8 Department of Medical Sciences, University of Torino, Italy; 9 Université Paris-Cité, Hôpital Beaujon, Service d'hépatologie, Assistance Publique-Hôpitaux de Paris, Clichy, France; 10 INSERM Unité Mixte de Recherche 1149, Clichy, France; 11 Universite Claude Bernard Lyon 1, CarMen Laboratory, INSERM, INRAE, Pierre-Bénite, France; 12 Hospices Civils de Lyon, Département Endocrinologie, Diabète et Nutrition, Hôpital Lyon Sud, Pierre-Bénite, France; 13 Cairo University Hospitals HIV Clinic, Department of Endemic Medicine, Faculty of Medicine, Cairo University, Egypt; 14 Department of Pediatric Gastroenterology and Hepatology, Ghent University Hospital, Department of Internal Medicine and Pediatrics, Ghent University, Ghent, Belgium; 15 Liver Unit, Hospital Clínic of Barcelona, Barcelona, Catalunya, Spain; 16 Institut d'Investigacions Biomèdiques August Pi i Sunyer (IDIBAPS), Barcelona, Catalunya, Spain; 17 Faculty of Nursing, University of Barcelona, Barcelona, Spain; 18 Center for Clinical Nursing Science & Department of Gastroenterology and Hepatology, University Hospital Zurich, Zurich, Switzerland; 19 Research Council (CNR), Institute of Clinical Physiology (IFC), Pisa, Italy; 20 Hepatology Research Unit, Department of Internal Medicine and Pediatrics, Ghent University, Ghent, Belgium; 21 Liver Research Center Ghent, Ghent University Hospital, Ghent, Belgium; 22 Newcastle NIHR Biomedical Research Centre, Newcastle upon Tyne Hospitals, NHS Foundation Trust, Newcastle upon Tyne, UK; 23 Translational and Clinical Research Institute, Faculty of Medical Sciences, Newcastle University, Newcastle upon Tyne, UK; 24 NIHR Newcastle Biomedical Research Centre, Newcastle upon Tyne Hospitals, NHS Foundation Trust, Newcastle upon Tyne, UK; 25 Department of Vascular Medicine, Amsterdam UMC, Amsterdam, the Netherlands; 26 Amsterdam Gastroenterology Endocrinology and Metabolism Institute, Amsterdam UMC, Amsterdam, the Netherlands; 27 Institute of Pathology, Medical University of Graz, Austria; 28 City University of New York Graduate School of Public Health and Health Policy (CUNY SPH), New York, NY, USA; 29 Barcelona Institute for Global Health (ISGlobal), Barcelona, Spain; 30 Faculty of Medicine and Health Sciences, University of Barcelona (UB), Barcelona, Spain; 31 La Mina Primary Care Academic Centre, Catalan Health Institute and Institute for Primary Health Care Research IDIAP Jordi Gol, Barcelona, Spain; 32 European Coalition for People Living with Obesity, Dublin, Ireland; 33 Institute for Cardiometabolism and Nutrition, Sorbonne Université and Hospital Pitié Salpêtrière, Paris, France; 34 Department of Endocrinology and Diabetology, Medical Faculty and University Hospital Düsseldorf, Heinrich-Heine-University Düsseldorf, Düsseldorf, Germany; 35 Institute for Clinical Diabetology, German Diabetes Center, Leibniz Center for Diabetes Research, Heinrich Heine University, Düsseldorf, Germany; 36 German Center for Diabetes Research (DZD e.V.), Partner Düsseldorf, Germany; 37 Stravitz-Sanyal Institute for Liver Disease and Metabolic Health, Div. of Gastroenterology, Hepatology and Nutrition, VCU School of Medicine, Richmond, VA, USA; 38 Division of Hepatology, Department of Medicine, Faculty of Health Sciences, University of Cape Town, Cape Town, South Africa; 39 Liver Unit, Regional Institute of Gastroenterology and Hepatology, Cluj-Napoca, Romania; 40 Department of Pathology, Aretaieion Hospital, Medical School, National and Kapodistrian University of Athens, Athens, Greece; 41 UCL Institute for Liver and Digestive Health, Royal Free Hospital and UCL, London, UK; 42 European Reference Network of Hepatological Diseases (ERN-RARE LIVER), Hamburg, Germany; 43 Dutch Liver Patients Association, Hoogland, The Netherlands; 44 Centre d'Investigation Clinique Plurithémathique Pierre Drouin and Département de Cardiologie, Institut Lorrain du Coeur et des Vaisseaux, CHU de Nancy, Nancy, France; 45 The Global NASH/MASH Council, Washington, DC, USA; 46 School of Public Health, University of Haifa, Haifa, Israel; 47 Fibrosis, Fatty Liver and Steatohepatitis Research Center Odense (FLASH), Department of Gastroenterology and Hepatology, Odense University Hospital, Odense, Denmark; 48 Department of Clinical Research, Faculty of Health Sciences, University of Southern Denmark, Odense, Denmark

**Keywords:** Steatotic liver disease, SLD, metabolic dysfunction-associated SLD, MASLD, MetALD, alcohol-related liver disease, ALD, management, guidance, EASL, multidisciplinary

## Abstract

Steatotic liver disease (SLD) is a spectrum of highly prevalent liver diseases encountered at many different healthcare levels. Awareness about metabolic dysfunction-associated SLD (MASLD), MetALD (MASLD and increased alcohol intake) and ALD (alcohol-related liver disease) is increasing beyond hepatology, as reflected by the various SLD guidelines issued by scientific societies from different disciplines, including endocrinology. The guidance presented here aims to improve cross-disciplinary knowledge and know-how by providing guidance to healthcare professionals from a global, holistic, multi-society and multi-stakeholder perspective. The patient trajectory was used as an overarching anchor point to ensure uniform guidance at all points along the SLD spectrum, with the ultimate goal of improving patient identification and optimising care. This consensus guidance covers patient identification and risk stratification, relevant cross-disciplinary primary and secondary care approaches, referral to and from tertiary care and strategies for disease management and monitoring.

## Graphical abstract

**Figure:**
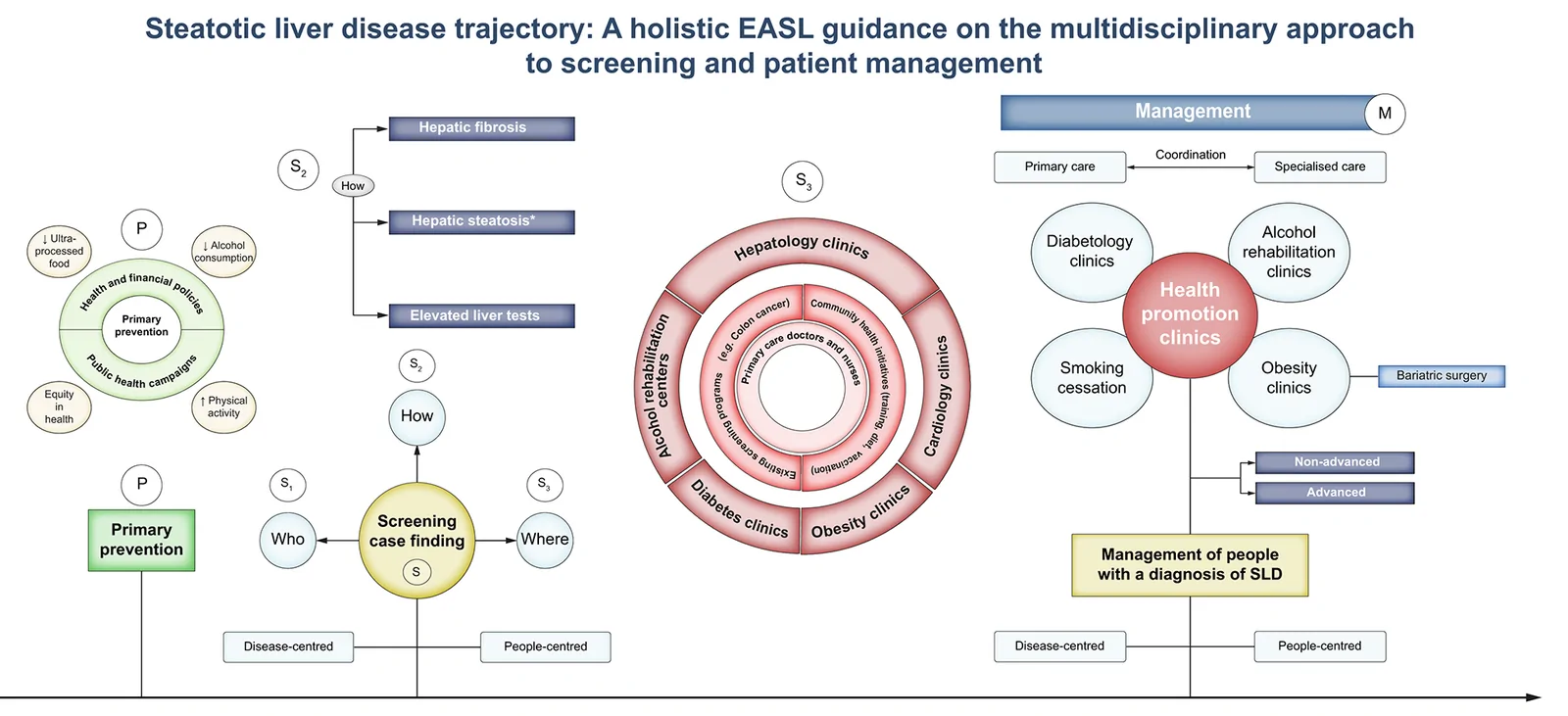

## Introduction

Steatotic liver disease (SLD) is a global public health threat, currently affecting more than 1-in-3 adults. SLD incidence and prevalence are increasing globally, largely driven by the rising prevalence of obesity and diabetes. At the same time, continued high alcohol consumption in Europe and increasing per capita intake in the rest of the world also contribute to the SLD burden.

Metabolic syndrome (MetS) components like obesity, hypertension, dyslipidaemia and insulin resistance increase the risk of many non-communicable diseases (NCDs), such as type 2 diabetes mellitus (T2DM), cardiovascular disease (CVD) and several types of cancer.^1^ Metabolic dysfunction-associated SLD (MASLD), defined as SLD with at least one cardiometabolic risk factor, is increasingly being recognised as a comorbidity and potentially severe complication of MetS.[1], [2], [3] Substantial (≥350 g/420 g [♀/♂] weekly) alcohol consumption that leads to alcohol-related liver disease (ALD) is another leading cause of liver-related mortality; a lower (<140 g/210 g [♀/♂] weekly) intake may accelerate MASLD, while moderate (140-350 g/210-420 g [♀/♂] weekly) consumption may act additively or synergistically with metabolic comorbidity to increase the risk of progressive liver disease or MetALD.^2^^,^^4^ Distinct and not discussed here is ALD with metabolic dysfunction (without SLD). MASLD, MetALD and ALD have led to an increased incidence of cirrhosis and primary liver cancer, with SLD now being the leading indication for liver transplantation in many regions globally.^3^

The relationship between SLD and metabolic dysfunction and/or MetS is multifactorial and often bidirectional. SLD is associated with metabolic dysfunction preceding MetS but may also reinforce or develop into MetS or its individual components.^1^ In susceptible persons, insulin resistance and/or chronic alcohol consumption lead to increased storage of hepatic triglycerides, lipotoxicity, inflammation, and hepatocyte injury, resulting in steatohepatitis, which over time triggers scar tissue formation (fibrosis).^2^ This can be progressive and may result in cirrhosis and acute-on-chronic liver failure. Liver fibrosis severity is the major determinant of liver-related prognosis and hepatocellular carcinoma (HCC) development, and the latter may develop even in the absence of advanced fibrosis or cirrhosis.^2^ While simple liver steatosis (*i.e*. fat accumulation in hepatocytes) may increase clinically apparent diabetes- and cardiovascular-related risk, liver-specific disease is mostly silent until its late stages.^3^ It is therefore important to proactively investigate potential liver complications in people living with risk factors who lack overt symptoms^4^

SLD-related problems have become more apparent at all healthcare and societal levels. Consequently, all healthcare professionals (HCPs), be it dietitians, exercise specialists, primary care doctors or secondary or tertiary care specialists, will encounter patients living with metabolic risk factors, who may or may not consume alcohol, and are thus at risk for SLD. Due to its multifactorial nature, the screening, diagnosis and treatment of SLD requires a multidisciplinary approach.^5^ To this end, many SLD guidelines have been developed by different scientific societies[6], [7], [8], [9], [10], [11], [12] and, for the first time, global research and action priorities have been developed to guide efforts against the disease.^13^^,^^14^ Nonetheless, guidance on SLD as a dynamic disease spectrum is limited.^15^

**Fig. 1:**
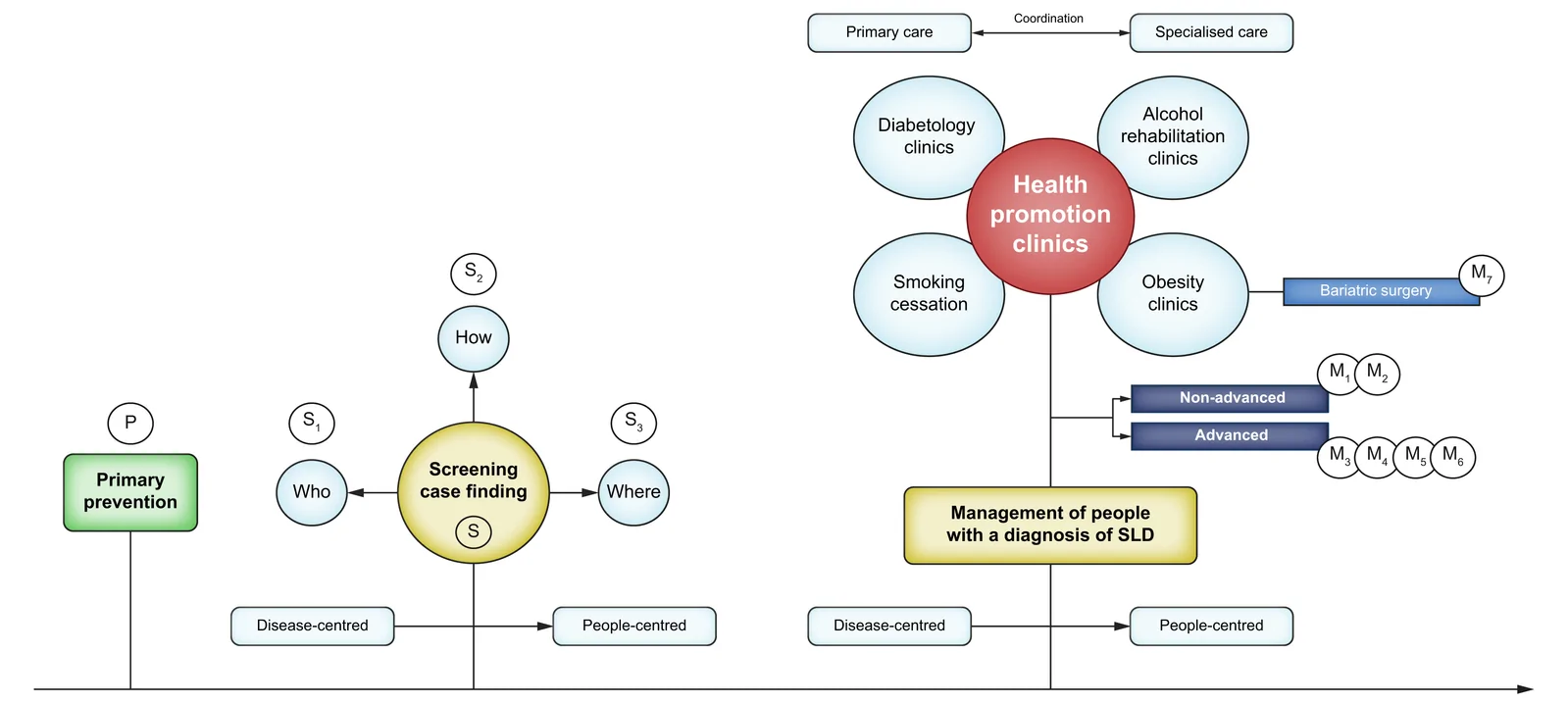
SLD trajectory summary. Primary prevention (P); Screening (S): Who (S1), How (S2), Where (S3); Management (M), Management of people living with SLD with non-advanced disease (M1), Lifestyle maganement (M2), Management of people living with SLD with advanced disease (M3), Management of people living with SLD with CSPH. (M4), Management of people living with SLD and ACLD (M5), Management of bariatric surgery in people living with SLD (M6-7). ACLD, advanced chronic liver disease; CSPH, clinically significant portal hypertension; SLD, steatotic liver disease.

The goal of this guidance is thus to provide recommendations for people at risk of developing, presenting with, or experiencing complications from SLD. The guidance covers prevention, risk stratification and care across different health systems (Fig. 1). This global, holistic, multi-society and multi-stakeholder guidance uses the patient trajectory as an overarching anchor point to cover all relevant areas and optimise patient identification and management, with the goal of improving health outcomes. The paper is divided into three main topics: 1) screening and case-finding strategies, 2) management of different SLD stages: how and who, and 3) advanced fibrosis and cirrhosis management. These topics are further divided into several subtopics.

## Methodology

This guidance was commissioned by the European Association for the Study of the Liver (EASL) based on a concept proposed by SF and AK. The process was designed by SF and approved by the EASL Governing Board in 2024. Two secretaries (WPB and LV) operationalised the process and were responsible for writing meeting reports and the interim and final versions of the protocol. Three multidisciplinary working groups, composed of a total of 36 panellists (Table S1), led by MT, JS and SF prepared statements and their justification. Statements were presented at the EASL SLD Summit in Estoril, Portugal, on 23 January 2025, and opened for public discussion and input. Statements were subsequently finalised, compiled and submitted to all panel members for approval.

## Screening and case-finding strategies

### Preventive and societal measures to combat liver disease

The high prevalence of metabolic risk factors and alcohol consumption, which together account for most liver-related morbidity and mortality,^16^ underscores the need for primary prevention of SLD and related liver fibrosis. Preventive actions include policy measures to reduce consumption of ultra-processed foods and alcohol, as well as public health campaigns and policies that promote healthy lifestyles incorporating physical activity, a balanced diet and avoidance of hazardous drinking,[17], [18], [19] along with economic and health policies to reduce health inequalities. Educational initiatives targeting the community and primary care to promote liver health may help reduce the burden of liver disease. Beyond this, social, cultural, economic, health policy and global trends that underlie the current pandemic of obesity and its related NCDs require a multifaceted approach to prevention, early detection and management at a global, national, regional and local health system level.

**Fig. 2:**
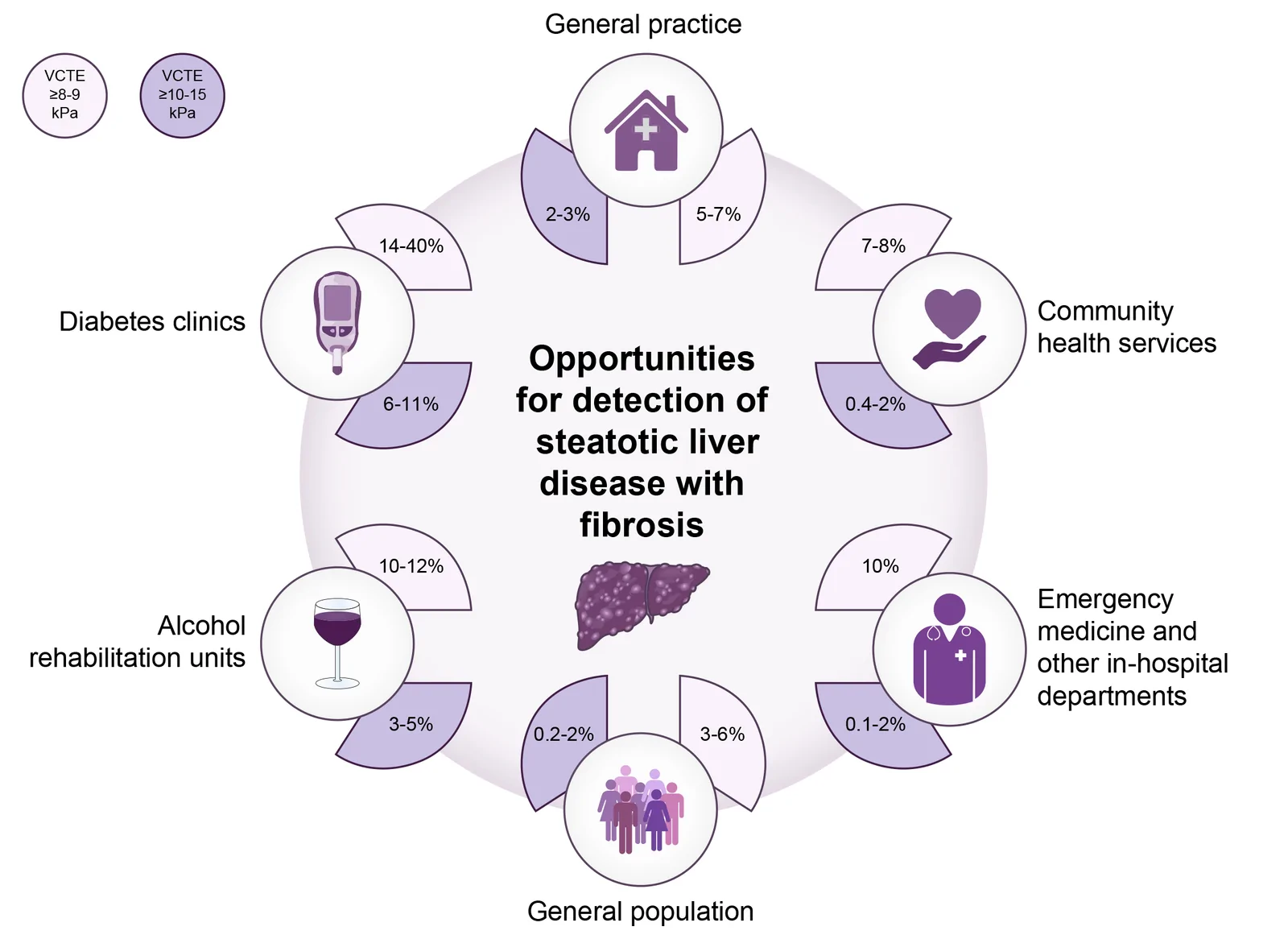
Opportunities for detection of possible significant fibrosis for (VCTE ≥8-9 kPa) and possible advanced fibrosis (VCTE ≥10-15 kPa) in different scenarios outside hepatology care. Primary care,^50^^,^^95^^,^^319^ community health services,^31^^,^^320^^,^^321^ diabetes and obesity clinics,^139^^,^^140^^,^[322], [323], [324] alcohol rehabilitation units,^46^^,^^101^^,^^103^^,^^325^ general population,^321^^,^[326], [327], [328], [329] emergency medicine and other in-hospital departments.^107^^,^^109^^,^^330^ VCTE, vibration-controlled transient elastography.

As for secondary prevention approaches, structured case-finding programmes that integrate non-invasive tests (NITs) for liver fibrosis identification may enable early detection of individuals living with SLD at high risk of progression to liver-related events (LREs).^20^ Appropriate subsequent management of these people may enable prevention of disease progression to cirrhosis and its complications, which may justify such screening in populations at risk (Fig. 2).

**Fig. 3:**
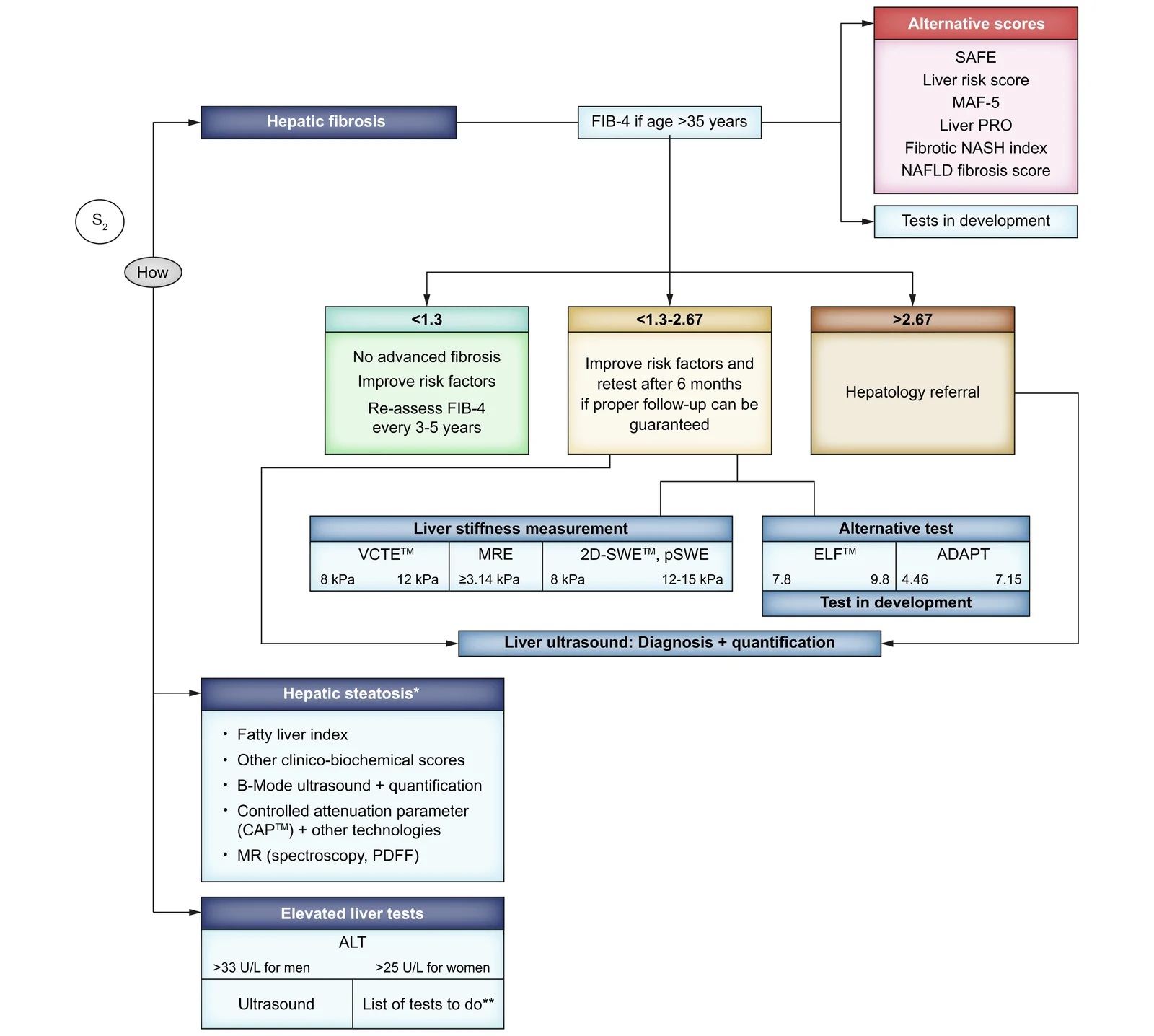
Screening, case finding (S2): how to screen. If age >65 years consider the lower FIB-4 cut-off of 2.0. The cut-offs to respectively rule out or rule in advanced fibrosis for VTCE^TM^, 2D SWE^TM^/pSWE, ELF^TM^ and ADAPT tests are reported. ∗Steatosis is always a signal of being metabolically unhealthy but does not always require liver-targeted management. It does require management of the CMRF. ∗∗List of tests to perform: hepatitis B surface antigen (HBsAg), hepatitis C virus (HCV) antibody, hepatitis A virus (HAV) antibody, hepatitis E virus (HEV) antibody, cytomegalovirus (CMV) antibody, Epstein-Barr virus (EBV) antibody, toxoplasma antibody, ferritin, transferrin saturation, alpha-1-antitrypsin level, caeruloplasmin, serum copper, anti-mitochondrial antibody, anti-smooth muscle antibody, antinuclear antibody, anti-liver-kidney microsomial (LKM) antibody, antineutrophil cytoplasmic antibodies (ANCA), coeliac antibodies, liver cytosol 1 (LC1) antibodies, soluble liver antigen/liver-pancreas (SLA/LP) antibodies, F-actin antibodies, Ro52 antibodies, glycoprotein (gp)210 antibodies, sp100 antibodies, serum immunoglobulins. ^§^The cut-offs can vary with different assays, *e.g*. a cut-off of ≥9 for significant fibrosis has been reported with Elecsys® ProC3 Cobas (Instructions for use 09088423500V2.0). 2D-SWE, two-dimensional shear wave elastography; ADAPT, age, presence of diabetes, PRO-C3, and platelet count; ALT, alanine aminotransferase; CAP^TM^, controlled attenuation parameter; ELF, enhanced liver fibrosis; FIB-4, fibrosis-4; Fibrotic NASH index, fibrotic non-alcoholic steatohepatitis index; MAF-5, metabolic dysfunction-associated fibrosis 5; MRE, magnetic resonance elastography; MR, magnetic resonance; NAFLD fibrosis score, non-alcoholic fatty liver disease fibrosis score; PDFF, proton density fat fraction; pSWE, point shear wave elastography; SAFE, steatosis-associated fibrosis estimator; VTCE, vibration-controlled transient elastography.

Screening for liver steatosis among the general population is currently not recommended, as isolated or simple steatosis does not confer a risk for LREs and there is no evidence to support general population-based fibrosis screening.^6^^,^^21^ However, the presence of liver steatosis remains clinically relevant, due to the associated increased risk of cardiometabolic disease. A Swedish epidemiological study of 10,568 individuals with biopsy-confirmed MASLD and 49,925 controls found that the presence of isolated steatosis alone was not associated with major adverse LREs, but resulted in an almost 70% increase in all-cause mortality.^22^ This provides an argument against screening for steatosis alone and supports screening for diagnostic targets associated with higher liver-related mortality, like steatohepatitis, or, more importantly, significant (≥F2) and advanced liver fibrosis (≥F3).

Guidance on case-finding for SLD-related liver fibrosis in primary, secondary and tertiary care, and setting details, which aligns with the global SLD research and action agendas^13^^,^^14^ and expert recommendations on comprehensive models of care^23^ are provided in the next section.

### Case-finding and risk stratification: Who, how and where

#### Targeting risk factors for optimised case-finding: “Who” (Fig. 1, panel S_1_)

##### Goals of non-invasive testing

Identification of liver fibrosis should be performed in those with an increased risk of liver disease. While case-finding is recommended, mass screening for SLD or liver fibrosis is not. When steatosis is detected, further assessment of the severity of liver disease, including steatohepatitis, fibrosis stage, and the risk of developing LREs, should be undertaken.

##### Who to target?

Often, a combination of medical history and clinical findings, including blood test results, will trigger an evaluation for fibrosis via NITs (Table 1).^6^^,^^9^ However, many HCPs outside of hepatology lack awareness about SLD and NITs.^24^ Furthermore, beyond evident cirrhosis, there is not a single key clinical finding to indicate the presence of fibrosis, leading to a recommendation of systematic case-finding or opportunistic screening in patients living with major risk factors: T2DM, obesity, including visceral obesity or adiposity, cardiometabolic risk factors (*i.e*. MetS) and hazardous drinking patterns.^6^^,^^9^ An increasing number of cardiometabolic traits is associated with a higher risk of advanced fibrosis, cirrhosis, HCC and all-cause mortality in individuals living with MASLD, MetALD and ALD.^12^^,^[25], [26], [27], [28], [29], [30], [31] Several demographic factors are associated with a higher risk of significant fibrosis, including age (typically ≥40–50 years), male sex and postmenopausal status, Hispanic ethnicity, and low socioeconomic status.^4^^,^^32^ Studies have also indicated that individuals >70 years living with SLD are not at a higher risk of liver-related mortality.^33^ Notably, the prevalence of liver fibrosis due to SLD is also rising among children, adolescents and young adults.^34^ This poses challenges for timely case-finding of liver fibrosis. Young adults may also be at increased risk of liver fibrosis, owing to their longer lifetime exposure to SLD. As alcohol is the strongest driver of fibrosis in SLD, an accurate assessment of alcohol intake history is mandatory during patient evaluation.^35^ Consumption of ≥1 standard drink per day (*i.e.* ≥14 g alcohol) in females and ≥2 standard drinks per day in males increases the lifetime risk of cirrhosis.^36^^,^^37^ Alcohol increases the risk of liver fibrosis, acting additively or synergistically with metabolic comorbidities in MASLD and MetALD, while SLD and alcohol independently and additively increase the risk of cirrhosis and all-cause mortality.^26^^,^^38^ One challenge is that alcohol consumption often follows a dynamic pattern in the short- and long-term, with periods of abstinence or low intake alternating with episodes of heavier intake.^39^^,^^40^ Therefore, beyond the current drinking pattern, HCPs also need to establish whether the person has a history of harmful drinking exceeding 1-5 years.^35^ Because alcohol consumption is under-reported in an estimated 25-30% of individuals living with MASLD, and even more so in clinical settings, it is essential that alcohol use is assessed using a triangulated approach.^41^ This should combine a thorough medical history, a validated questionnaire, such as the Alcohol Use Disorders Identification Test (AUDIT, 10 questions) or AUDIT-C (first 3 questions of AUDIT), and a phosphatidylethanol (PEth) measurement.^42^ Given the dynamic nature of alcohol intake, such assessments should be repeated over time to ensure an accurate diagnosis.^43^

**Table 1:** List of risk factors for SLD that should trigger non-invasive fibrosis testing.

**Patient history**	

Known metabolic dysfunction	T2DM, insulin resistance, dysfunctional adiposity, dyslipidaemia, arterial hypertension, PCOS
Family history	1st degree relatives with a history of cirrhosis, HCC or T2DM∗
Information from medical history indicating risk of MetALD or ALD	Report of harmful use of alcohol: Daily drinking, frequent heavy episodic drinking, drinking more than 7/14 units per week (female/male) for at least 1-5 years, being hospitalised with AUD diagnoses
Information from medical history indicating risk of MASLD	Sleep apnoea, weight gain, a high-sugar (especially sugared beverages), high fat and -calorie diet, sedentary lifestyle
Symptoms indicative of advanced chronic liver disease∗∗	Fatigue, itching, weight loss, muscle wasting, malaise, abdominal pain, gynecomastia, abdominal distension, generalised pain, haematomas, sleep disturbances, appetite loss, nausea

**Clinical findings**	
Obesity (dysfunctional adiposity)	BMI ≥30 kg/m^2^, BMI ≥25 kg/m^2^ and waist-to-height ratio ≥0.50, waist circumference >94/90 cm for Europid/Asian males or >88/80 cm for females, waist-hip-ratio >0.90 for males or >0.85 for females
Ultrasonography	Hyperechoic liver parenchyma, rounded liver edges, ultrasound beam attenuation, heterogenous parenchyma and narrowed intrahepatic vessel walls, lobular surface, slow portal venous flow (<15 cm/s), ascites, splenomegaly (>12 cm), collaterals
External signs of cirrhosis and portal hypertension	Palmar erythema, gynaecomastia, spider angiomas, hypogonadism, muscle wasting
Blood test results	
Hepatic inflammatory activity	Elevated (>ULN) ALT, AST, GGT, ferritin
Cholestatic injury∗∗∗	Elevated (>ULN) alkaline phosphatase, GGT, bilirubin
Portal hypertension	Low platelet count, suspected on imaging
Liver function	Low albumin, high INR and bilirubin

ALD, alcohol-related liver disease; ALT, alanine aminotransferase; AST, aspartate aminotransferase; AUD, alcohol use disorder; BMI, body mass index; GGT, gamma-glutamyl transferase; HCC, hepatocellular carcinoma; INR, international normalized ratio; MASLD, metabolic dysfunction-associated SLD; MetALD, MASLD and increased alcohol intake; PCOS, polycystic ovary syndrome; T2DM, type 2 diabetes mellitus; ULN, upper limit of normal.

∗ Consider performing NIT on a case-by-case basis.

∗∗ If not known to be living with chronic liver disease.

∗∗∗ Based on a combination of factors, *e.g*. an isolated elevated ALP or GGT may be related to disease other than liver disease, such as bone disease or disturbances in the (para)thyroid axis and/or calcium regulation. Choose NIT on a case-by-case basis.

Notably, although the new MASLD and MetALD definitions suggest that moderate alcohol use may not be harmful, recent studies show that this should be interpreted cautiously. In one study, those living with MASLD and one cardiometabolic criterion who consumed a moderate (10-13/10-20 [♀/♂] units weekly) amount of alcohol had a 13% risk of significant fibrosis and an increased risk of metabolic dysfunction-associated steatohepatitis (MASH), with these risks increasing to 25% and 51%, respectively, among those living with 4-5 cardiometabolic traits. Thus, drinking any amount of alcohol should be seen as potentially hazardous.^31^ This seems to be especially true for younger adults, who have the potential for a longer exposure to risk factors and may therefore be at higher risk of developing severe liver disease^44^

#### Non-invasive testing in different settings and resources: “How” (Fig. 3)

##### Main types of NITs

NITs are increasingly used for SLD-related liver fibrosis screening due to their cost-effectiveness, safety, and greater accessibility than the reference standard of liver biopsy. The usability of liver biopsy is limited due to the risk of pain, complications, sampling error, observer variability, availability of staff with appropriate expertise and costs.^6^ The three main types of NITs are composite scores, serum biomarkers and imaging.

Depending on the setting and availability of resources, NITs can be used for liver fibrosis and steatosis/steatohepatitis risk assessment. Importantly, NITs also have prognostic value across the spectrum of MASLD, MetALD and ALD. In practice, NITs are primarily used to assess fibrosis, as a risk-stratifying strategy, and their use as direct prognostic markers aligns with this. Important metrics to consider when analysing NITs largely depend on the context of use (*e.g.* is it being used to exclude advanced liver disease in a low-prevalence population or to identify advanced liver disease in a high-prevalence population?). Ideally, a NIT would accurately identify individuals with and without liver disease, with high sensitivity and specificity, and correspondingly high negative and positive predictive values (NPV and PPV). Notably, NIT performance is strongly influenced by background disease prevalence and severity in the target population.^45^ A NIT used in a tertiary care setting cannot simply be applied in a primary care or population-based setting without rigorously testing it in that specific context. In a screening setting, the primary NIT must be sensitive enough to detect liver disease with a high NPV. In a two-tier strategy, a secondary test with a high specificity and PPV would then be applied. Two-tiered strategies are currently being evaluated.^46^

##### Liver disease assessment in primary care and community healthcare services

• Must-have

*Liver fibrosis detection*

In primary care, a good initial liver fibrosis screening test would be a simple serum-based NIT. Index tests should have a high NPV to exclude liver fibrosis. For example, the presence of significant liver fibrosis, defined as a liver stiffness measurement (LSM) ≥8 kPa, in a population-based setting ranges from 3-10% and largely depends on cardiometabolic risk factors (Table 1).^4^^,^^47^^,^^48^ Without using a NIT, this already means a NPV of 90-97%. Small increases in the NPV will lead to a better exclusion of people who do not have fibrosis, a lower number of undetected fibrosis cases, and a reduction in unnecessary referrals, reducing the burden on already strained healthcare systems. However, the NPV is only one of many important metrics of a diagnostic test. It is not only important to correctly exclude, but also correctly detect, individuals living with liver disease, via a high specificity and PPV.

Guidelines recommend screening individuals with metabolic risk factors to assess their fibrosis risk, more precisely their risk of significant or advanced fibrosis, corresponding to a histological fibrosis (F) stage of ≥F2 or FibroScan® LSM value ≥8 kPa per the non-alcoholic steatohepatitis (NASH)/MASH Clinical Research Network Scoring System.^6^ The Fibrosis-4 (FIB-4) index is also recommended^6^^,^^9^^,^^49^ to rule in advanced fibrosis (>2.67) or rule it out (<1.3 or <2.0 among those >65 years).^6^^,^^9^

While the current guideline recommendations should be followed, we provide key additional comments in light of important new and emerging evidence since their publication. Although FIB-4 is the most widely used index, it has important limitations. FIB-4 only slightly increases the NPV compared to no test at all, retains a low area under the curve of 0.55-0.70 for the detection of significant or advanced liver fibrosis, and is primarily driven by age, leaving younger populations of <40 years underserved.[50], [51], [52] In older individuals, the referral rate using this test increases significantly. This led to the development of age-adjusted cut-offs (*e.g*. for individuals >65 years), making the test more complex to use.

To further improve early case finding, a wealth of new NITs for population-based settings have been developed, such as the Steatosis-associated Fibrosis Estimator score,^48^^,^^53^ LiverRisk score,^52^^,^^54^ metabolic dysfunction-associated fibrosis-5 score,[42], [43], [44], [45] LiverPRO score,^55^ Fibrotic NASH Index,^56^ and NAFLD (non-alcoholic fatty liver disease) fibrosis score,^57^ among others.^58^ It is expected that some or many of these may be implemented in future guidelines, depending on the setting, regulatory status and cost, and possibly via multi-tier diagnostic algorithm approaches.^58^ For instance, The National Institute for Health and Care Excellence guidelines recommend the more specialised and proprietary Enhanced Liver Fibrosis (ELF) test for use in primary care.^59^^,^^60^

Importantly, emerging evidence across the SLD spectrum consistently demonstrates a very low liver-related risk at an LSM <10 kPa in hospital-based settings.^61^ In a context of increasing awareness and broader assessment, adopting an LSM threshold higher than ≥8 kPa could meaningfully reduce the burden of false positives. Nonetheless, it should be noted that individuals with an LSM ≥8 kPa are at risk of developing advanced liver disease and, as such, should be targeted for primary and secondary prevention. Moreover, individuals with an LSM ≥8 kPa but <10 kPa may follow the current clinical care pathway and be reassessed within 1-3 years, allowing for those whose disease progresses fast to be captured.^6^^,^^61^ The balance of keeping people with such LSM values in primary care *vs.* referring them to hepatology should be carefully considered and weighed against the overall burden of liver disease and the strain on healthcare systems. Economic evaluations are needed to assess the costs and benefits of different referral strategies.

*Assessment of alcohol use*

Beyond a thorough clinical interview, the use of quantity-frequency questionnaires is recommended to assess current alcohol use^8^ In the 3-item AUDIT-C questionnaire, ≥3/4 points for females/males suggests harmful alcohol use, while ≥8 points suggests dependence.^62^ The Quick Drinking Screen is another short tool which addresses past 90-day drinking, compared to past year intake as assessed by the AUDIT-C.^63^ Longer alternatives are the 25-item, binary Michigan Alcohol Screening Test, or the 10-item AUDIT.^64^^,^^65^ The gold standard is the calendar-based timeline follow-back method, which estimates daily alcohol consumption ranging from 7 days to 24 months prior to the assessment.^66^

• Nice-to-have

*Liver fibrosis detection*

Despite its high diagnostic accuracy in clinical settings, the use of vibration-controlled transient elastography (VCTE) for screening advanced liver fibrosis in primary care or specialised clinics remains dependent on further validation, as well as local availability and expertise.[67], [68], [69] An interesting alternative could be the ultra-portable point-of-care two-dimensional transient elastography (2D-TE) devices that have recently emerged in clinical use, but these still need validation^70^

*Non-invasive biomarker assessment of alcohol use*

Direct biomarkers of alcohol use can support self-reported alcohol consumption. These biomarkers estimate recent, not historical, alcohol intake, over the following time frames: ethanol in serum, urine or breath (hours); ethylglucuronide or ethylsulfate in urine (a few days); PEth in blood (a few weeks); and ethylglucuronide in hair (a few months).^71^ Except for ethanol measurements, direct biomarkers are products of non-oxidative pathways, whereby ≤1% of alcohol is metabolised. Consequently, a positive measurement rules in any recent exposure to alcohol. Yet, these markers are only moderately calibrated to drinks per day due to considerable inter-individual variation.^72^ This variability reflects differences in daily alcohol intake, activity of the enzymes that metabolise the alcohol biomarkers, and ethanol concentrations, which are influenced by factors like body composition, sex and whether or not alcohol is consumed on an empty stomach.^73^ Due to these variations, such biomarkers are best suited to rule out recent alcohol consumption and rule in heavy drinking, while in-between measurements can be difficult to interpret. PEth is the most widely used direct alcohol biomarker. A concentration <20 ng/ml (0.03 μmol/L) denotes occasional, low-level drinking, while one ≥200 ng/ml (0.28 μmol/L) indicates excessive drinking. Furthermore, a study in 222 individuals living with chronic liver disease found that a PEth ≥80 ng/ml (0.11 μmol/L) detected past month drinking of ≥4 daily drinks, with a high sensitivity but suboptimal specificity. This aligns with a large population study in Norway, where a PEth cut-off of 86 ng/ml indicated ≥4 daily drinks, with an 80% sensitivity; a PEth ≥40 ng/ml showed a high sensitivity for ≥1 daily drink.^42^^,^^74^^,^^75^

*SLD detection*

In general, guidelines do not recommend population-based SLD screening, but it may be helpful to diagnose or assess the risk of SLD as it is a sign of the onset of the disease and a marker of ectopic lipid accumulation, which denotes a metabolically unhealthy state. Abdominal ultrasound can be useful for the diagnosis of steatosis. In primary care, steatosis is usually found incidentally on an abdominal ultrasound that was performed for another reason.^6^ Another way to diagnose steatosis is by using the Fatty Liver Index, although its applicability may be limited.^76^^,^^77^

##### NITs in secondary care

• Must-have

*Confirmation of hepatic steatosis*

In secondary care, abdominal ultrasound is recommended to confirm steatosis in individuals at higher risk of SLD, such as those attending substance use, diabetes, or cardiology clinics. Abdominal ultrasound detects SLD with a 60-94% sensitivity and 66-97% specificity,^78^ despite a lower performance at mild degrees of steatosis.^79^ A semi-quantitative abdominal ultrasound-based approach scores liver brightness (mild/moderate or severe), the presence of acoustic attenuation, vessel blurring and focal sparing, and the level of difficulty in visualising the gallbladder wall or diaphragm.^80^ Neither the calculation of the hepato-renal index, nor that of the controlled attenuation parameter, are currently recommended for steatosis screening^81^ However, ultrasound fat quantification methods are increasingly available on a larger variety of machines, showing a 76% sensitivity and 84% specificity to detect any grade of steatosis and having better accuracy compared with the controlled attenuation parameter for each grade of steatosis^81^^,^^82^ Therefore, if available, ultrasound attenuation coefficients could be used alongside abdominal ultrasound to detect steatosis. Ultra-portable devices for point-of-care steatosis quantification are also becoming available, opening the door for steatosis screening in at-risk populations.^83^

*Detection and diagnosis of liver fibrosis*

The prevalence of liver fibrosis is higher in secondary care and outpatient settings, ranging from 15-28%. Depending on resources and the setting, NIT serum biomarkers should be used as a first-line test (as in the previous section for primary care) if not done so already. This will assess the need for further liver-health check-up.^84^

Two widely used NITs for this latter purpose are either an LSM or a second-line serum test such as ELF, which is approved by the FDA and has a CE marking in Europe. LSM can be performed by VCTE (FibroScan®) and two-dimensional shear wave elastography (2D-SWE), both of which provide relatively accurate and reproducible assessment of liver fibrosis in secondary care. Among elastography techniques, VCTE is the most validated and most widely used. LSM <8/>12 kPa accurately excludes/confirms advanced fibrosis.^84^ Ultrasound-based 2D-SWE has the advantage of direct visualisation of the liver (also offering the possibility to detect signs of advanced liver disease – irregular liver margins, collaterals, ascites or splenomegaly), but the disadvantage of different cut-off values dependent on the manufacturer. Shear wave elastography (SWE) can be used to rule out (<8 kPa) and rule in (>12–15 kPa) advanced liver fibrosis in individuals with MASLD.

For hepatology clinics, this assessment may be regarded as standard of care and therefore as a “must-have”; others should refer patients based on their risk to clinics using these elastography-based tests. Nevertheless, these findings should be interpreted with caution, given their reduced accuracy in individuals living with obesity.^85^ In addition, elastography can overestimate the degree of liver fibrosis in patients with active inflammatory activity and high liver enzyme levels. VCTE has been the most extensively validated. Other imaging-based methods include 2D-SWE and ARFI (acoustic radiation force impulse), the latter requires only standard equipment which makes it a good choice in low-resource settings, yet findings are operator dependent. Also, a high variability of around 30-35% and high risk of false-positive results has been reported with several elastography techniques (different day, different operator),^86^ which implies that no firm conclusions can be drawn based on one elevated value and an average of at least two different measures (different time points) is recommended.

As a proprietary second-line serum test, the ELF test has been most extensively validated, has achieved FDA approval as a prognostic test for MASH, has received CE marking in Europe, and has been incorporated into guidelines.^6^^,^^9^^,^^59^

Previous analyses have shown that a sequential use of NITs with either VCTE or the ELF as a second-line test can increase the diagnostic accuracy for fibrosis staging and outcome prediction. More importantly, higher values of LSM and ELF are associated with an increased risk of hepatic- and extrahepatic adverse outcomes and all-cause mortality and hence serve as direct prognostic markers rather than surrogates for fibrosis.^9^ According to LSM, patients can be further assessed regarding the risk of compensated advanced chronic liver disease (cACLD) and the probability of clinically significant portal hypertension (CSPH).^87^ Based on the results of VCTE or ELF, treatment recommendations can be given for either referral back to primary care or even tertiary care if liver biopsy is needed, *e.g*. for ruling out comorbid liver diseases other than SLD.

##### NITs to detect liver fibrosis in tertiary care

• Must-have

In specialised hepatology clinics, the fibrosis score suspected by the index test needs to be confirmed by either complex serum tests, or by VCTE as previously mentioned in the section on secondary care. Complex serum tests usually assess various extracellular matrix proteins involved in fibrinogenesis and fibrinolysis. The most validated test to confirm/exclude advanced fibrosis is the aforementioned ELF score >9.8.^6^ The ADAPT score is an interesting alternative awaiting large-scale validation.^88^ Other potential complex biomarkers include FibroTest, Hepascore and FIBROspect-II (FS-II), pro C3, and NIS2+, among others.

Concerning VCTE, if LSM is >15 kPa the probability of cACLD is high. The rule of 4 can be used to interpret ARFI-SWE techniques and to rule out/in cACLD (<9 kPa and >13 kPa, respectively). In this setting, the Baveno VII criteria apply to stratify the risk of CSPH and LRE and are described in more detail later in this document.

• Nice-to-have

A further third-line approach includes magnetic resonance elastography (MRE), which is more sensitive and specific than the other non-invasive tests, with less operator variability, and has the ability to assess for liver fibrosis complications. However, its use should take into account patient and healthcare system resources, as it is more costly than other imaging modalities and is not widely available.^89^

##### Role of liver histology in compensated SLD – secondary or tertiary care

Current guidelines by leading scientific societies recommend liver biopsy and evaluation of liver histology in selected individuals with compensated SLD to help guide patient management in several settings.^6^^,^^9^^,^^11^^,^^12^^,^^90^ Histology has an important role in establishing competing aetiologies of liver disease.

(1) *If the cause(s) of liver disease is/are uncertain:*

Individuals who are diagnosed with SLD based on a combination of clinical, biochemical and radiological methods may have, beyond metabolic dysfunction and/or alcohol, alternative or additional causes of liver disease that cannot be detected or ruled out with certainty by non-invasive methods. Liver diseases that may present with liver test abnormalities similar to SLD and can lead to fibrosis include autoimmune hepatitis, primary biliary cholangitis, small-duct primary sclerosing cholangitis, sarcoidosis, vascular or hereditary liver diseases, and drug-induced liver injury. Except for ALD, which in the early stages shares a broad morphological overlap with MASLD, all other liver diseases considered in the differential diagnosis are characterised by typical histological changes that are distinct from those in MASLD.

(2) *If the stage of liver disease cannot be assessed with certainty:*

Several factors may influence non-invasive fibrosis assessment by transient elastography including ascites, inflammation of the liver parenchyma, cholestasis, congestion, central adiposity and anatomical aberrations. In addition, non-invasive markers of liver fibrosis may show discrepant results with respect to the presence of severe fibrosis; in such cases, liver biopsy is appropriate to determine the histological fibrosis stage. Steatohepatitis can only be diagnosed with a liver biopsy. Confirmation or exclusion of advanced fibrosis/cirrhosis is an important determinant to guide patient management.

(3) If one/more liver tumours are found, that cannot be diagnosed with certainty:

MASLD is associated with hepatic adenomas, which carry a risk of development to HCC. Therefore, all patients with space-occupying lesions should be referred to hepatology for further assessment. In these settings, having an MRI or CT scan is a must-have.^6^^,^[91], [92], [93]

If these tumours cannot be characterised with certainty by non-invasive methods or if the tumour(s) is/are smaller than 1 cm in size in the presence of cirrhosis, biopsy of the tumour, preferentially including a biopsy of the non-tumoural liver tissue, may be necessary to diagnose the precise nature of the liver tumour and other relevant characteristics of the underlying liver disease. In general, biopsy of a liver tumour is often only recommended after multidisciplinary discussion.^94^ Non-invasive criteria for the diagnosis of HCC only apply in the setting of cirrhosis, making biopsy of a suspected lesion in the non-cirrhotic liver necessary.

#### Opportunities and different healthcare settings for case-finding: “Where” (Fig. 1, panel S_3_)

##### General practitioners

Opportunities for early liver risk stratification are abundant, and very relevant in primary care. General practitioners and nurses managing diabetes and obesity care are ideally positioned to identify and manage the population at risk of LREs, as they already treat and monitor individuals living with obesity, T2DM, dyslipidaemia, arterial hypertension, or excessive consumption of alcohol.^95^^,^^96^ Moreover, primary care professionals also have a relevant role in case-finding among individuals with alcohol-related problems. This makes liver fibrosis risk assessment and liver health discussions a fitting extension of established general practitioner care.^97^ As a practical example, incorporating a liver health check-up that includes a NIT to assess liver fibrosis risk would be easy to implement in most clinics where patients undergo a yearly check-up for T2DM and/or cardiometabolic disorders.^98^

##### Community health services

Community health initiatives such as exercise, diet or vaccination programmes, and municipal alcohol rehabilitation centres, may also reach populations less likely to seek traditional care due to stigma, lack of awareness, or low health literacy. In these initiatives, it is important to ensure a link between social and health services and to facilitate access to primary care whenever required. Case-finding is especially important in deprived and vulnerable populations to reduce inequality in health.^99^

##### Addiction units

Addiction units provide a unique setting to screen for ALD, as patients who visit alcohol rehabilitation services are usually highly motivated to change. Early detection of ALD is particularly important as these individuals are generally diagnosed at more advanced liver disease stages than individuals with liver disease from other aetiologies.^100^ Consequently, the alcohol use disorder (AUD) rehabilitation setting is a very important opportunity to increase awareness of liver damage, ensure ALD management, and support long-term abstinence.[101], [102], [103] In a systematic review of 15 studies investigating liver disease detection among individuals with AUD, ALD fibrosis detection rates ranged from 5.6% to 81%.^104^ The high heterogeneity was mainly driven by setting: acute hospital admission to addiction care yielded cirrhosis rates of 11–30% cirrhosis, whereas community-based AUD care reported rates of 1.2–2.3%.^104^ In the largest screening study to date, including 1,850 participants from the general population with a self-reported history of long-term excess alcohol consumption, 10% had LSM ≥8 kPa by VCTE.^103^

##### Endocrinology, cardiology, obesity and diabetes clinics

Specialised endocrinology, cardiology, diabetes and obesity clinics can follow a similar model to primary care and offer another critical opportunity, given the high prevalence of liver fibrosis among individuals living with metabolic syndrome. Here, a well-defined framework already exists for annual screening of eye, cardiovascular, renal, and neurological complications of T2DM, making a strong case for including systematic liver risk stratification in routine follow-up visits.^7^^,^^98^ According to a systematic review including 28 observational studies of more than 10,000 people, 20% of individuals with T2DM have elevated LSM, with most studies using the 8 kPa cut-off.^105^ A prospective multi-centre study including patients with T2DM or obesity followed in diabetology and nutrition clinics reported that 18% of patients engaged in systematic screening for advanced fibrosis require specialised care in hepatology.^106^ Indeed, obesity and endocrinology societies have issued joint guidelines advocating screening for SLD and liver fibrosis in individuals at (cardio)metabolic risk.^7^

##### Other hospital-based settings

Individuals with SLD, especially those with undiagnosed cirrhosis, engage more frequently with in-hospital care than individuals without chronic liver disease.^107^^,^^108^ Emergency rooms, internal medicine, orthopaedics, and surgical departments provide natural opportunities to incorporate liver testing into investigations. By embedding liver risk assessment into existing protocols for conditions like cardiovascular events, accidents, fractures, and infections, these departments may flag patients for liver testing when the acute condition is stabilised. However, it should be stressed that all of the NITs discussed should be used in a chronic stable clinical state; therefore, it is important NITs be repeated when conditions are stable. The key challenge is poor retainment of patients after discharge, and the poor validity of most NITs in acutely ill patients.[109], [110], [111] Patients deemed at high risk according to such evaluation should be referred to the hepatologist and assessed when they reach a more stable condition.

##### Improvement of healthcare-seeking behaviour and reduction of stigma

Reducing perceived stigma and improving healthcare-seeking behaviour is crucial.^112^ HCPs should adopt patient-centred communication strategies to discuss liver health sensitively, such as using non-judgmental language and emphasising the shared goal of improving overall well-being. By asking open-ended questions, HCPs can create an opening for further evaluation, fostering trust and reducing stigma.

##### Call for transition to holistic people-centred approaches

Socio-economic inequities among people living with SLD exist at all intervention levels, from hospital-based to population-based.^110^^,^^111^ The World Health Organization calls for a more precise focus to reach the most affected and at risk to address inequities, yet this is often not the case for SLD, which is under-recognised and under-addressed by most health systems and by WHO itself.^112^ It is well established that low socio-economic status is associated with a higher MASLD and liver cancer prevalence. To address this, we need a transition from disease-centred healthcare systems to holistic, people-centred care within integrated health systems. For example, by incorporating "metabolic dysfunction" into the the definition of MASLD, some of the hardest to reach will be identified within hospitals, where many are already diagnosed with T2DM or CVD. However, a broader approach is needed in which the norm is disaggregated analyses of data by sex, age and other relevant population characteristics to track and monitor inequities and ensure that the most affected and at-risk populations are not left behind. Countries should continue pushing for universal health coverage and design better integrated, interprofessional, person-centred service delivery models, which can contribute to a paradigm shift in health systems’ approach to care and service delivery.

### Different scenarios for management of patients at high risk of fibrosis after targeted screening in primary care

How to communicate – visually and verbally – a high estimated risk for clinically relevant liver disease to a person at high risk for liver fibrosis in a general population or primary care setting remains challenging. This is an important issue because, on a population level, NITs may yield false-positive or false-negative results due to low disease prevalence and their performance characteristics. Not all the drivers of SLD and subsequent hepatic fibrosis are modifiable, and those that are may not be easy to change.^113^ Therefore, the diagnosis of a high-risk score should include a comprehensive, integrated package of care (Box 1).^114^^,^^115^

Box 1 **Overview of integrated package of care.**

Confirmation of hepatic fibrosis by VCTE, SWE, ARFI, advanced imaging techniques like MRE, or proprietary direct marker-based scores like the ELF and other advanced NITs, or ultimately liver biopsy according to previously listed indications in specialised hepatology clinics.

Lifestyle interventions, including tailored healthy diet, alcohol and physical activity advice, addressing mental health issues and sleep quality. Can be done by multidisciplinary primary care teams or Health Promotion Centres.

Alcohol consumption evaluation, AUD diagnosis and referral to AUD centre for treatment.

Pharmacotherapy using “old” drugs (*e.g.* statins) or newly developed or repurposed drugs like GLP-1RAs, dual GIP/GLP-1RAs, SGLT2 inhibitors (according to the approved indications as T2DM and obesity), and specific liver-targeted drugs, such as resmetirom, where available. Statins should be used when available and may also confer hepatic benefit. Re-evalua-tion and possible cessation of medication used by patients known for causing SLD or fibrosis.

Management of comorbid conditions including T2DM, obesity, obstructive sleep apnoea syndrome, (chronic) kidney disease, with appropriate cardiovascular disease prevention and cancer screening.

General health-related interventions include evaluating and managing malnutrition, sarcopenia and frailty. Employment of frailty indicators, such as the Liver Frailty Index. In addition to advice and access to vaccinations, such as HAV, HBV, and shingles.

Liver health-related interventions, including beta-blockers and/or endoscopy for those with indications of CSPH criteria, management of decompensation events, including access to liver transplantation, HCC surveillance and regular follow-up.

In high-resource settings, this care package can be more easily provided considering the concept of precision medicine while tailoring a patient’s package and ensuring a well-structured multidisciplinary team including a hepatologist, gastroenterologist, diabetologist, surgeon and allied health professionals (*e.g.*, dietitian, exercise specialist, psychologist and social worker).^116^

AUD, alcohol use disorder; ARFI, acoustic radiation force impulse; CSPH, clinically significant portal hypertension; ELF, enhanced liver fibrosis; GLP1-RA, glucagon-like peptide-1 receptor agonists; GIP/GLP1-RA, dual gastric inhibitory polypeptide/GLP1 receptor agonists; HAV, hepatitis A virus; HBV, hepatitis B virus; HCC, hepatocellular carcinoma; HPC, health promotion centres; MRE, magnetic resonance elastography; NIT, non-invasive test; SLD, steatotic liver disease or fibrosis; SGLT2, sodium-glucose transporter 2; SWE, shear wave elastography; VCTE, vibration-controlled transient elastography.

Alt-text: Box 1

### Low-resource settings and socioeconomic disparities in SLD

In low-resource settings, lessons learned from other public health threats like the COVID-19 pandemic and human immunodeficiency virus (HIV) should be applied.^117^ Liver diseases are associated with high levels of stigma and discrimination due to their links to alcohol use, obesity and viral hepatitis B and C. Community leadership and empowerment with appropriate knowledge and resources are crucial to maintaining linkage to care among individuals identified as high risk. Trained peer supporters and community workers can lead lifestyle interventions, utilising their understanding of contextual factors including food quality and security, available and acceptable physical activity, economic stability, and social context. For example, some local communities consider obesity and physical inactivity to be signs of good health and wealth.

The Reuters Institute Digital News Report 2023 highlighted that several African nations have over 25 million TikTok users, indicating well-established access to the internet and smartphones in these countries.^118^ This information emphasises the potential for telemedicine, particularly in addressing multidisciplinary management despite the shortage of local expertise in certain specialties. Telemedicine can serve as a valuable resource for supporting low-resource settings while also enabling the task shifting of care to primary care physicians and nurses by connecting them to local hepatology experts. Project ECHO programmes have been successfully used to deliver HIV and viral hepatitis care in under-resourced regions in Africa.

Point-of-care testing (*e.g.* ultrasound, transient elastography, and open-source non-invasive scores for advanced fibrosis and conditions like fibrotic MASH [such as the FAST score]) has improved the ability to reach underserved populations in various settings. In addition, point-of-care testing facilitates the co-localisation of hepatology services at endocrinology and bariatric clinics as a novel integration model. Another important benefit of simple algorithms and scores is their cost-effectiveness.^70^ For example, the PLEASE score for HCC risk stratification can help identify individuals who need more frequent and costly surveillance.^119^

#### NCDs in Sub-Saharan Africa

ub-Saharan Africa (SSA), a low- and middle-income region, has diverse and evolving economies and is experiencing increasing urbanisation alongside dietary and behavioural changes that may promote MASLD. Besides the burden of infectious diseases like tuberculosis, malaria, and HIV, there is an increasing burden of non-communicable diseases (NCDs), particularly a rising prevalence of hypertension, obesity and T2DM, which require integrated care.^120^ The all-age total disability-adjusted life-years due to NCDs increased by 67% between 1990 (90.6 million; 95% uncertainty interval 81.0–101.9) and 2017 (151.3 million; 133.4–171.8).^121^^,^^122^ This increase is driven by overlapping challenges of food insecurity, nutritional transition, and an associated increased consumption of calorie-dense foods and more sedentary urban lifestyles. NCDs among people living with HIV in SSA are also increasing and Africans with NCDs are on average >10 years younger than their counterparts in other world regions, with twice the risk of NCD-related mortality.^122^^,^^123^

The burden of NCDs in all four SSA regions is higher than the global average and SSA is anticipated to experience the largest global increase in NCD-related mortality.^121^^,^^124^

In SSA, the management of these NCDs, particularly T2DM and hypertension, occurs predominantly in the setting of primary care and nurse-driven community-based clinics. Increasingly, decentralised healthcare systems within the community are being developed to manage comorbidities and multi-morbidities (diabetes and hypertension) together with HIV. It is essential to develop simplified diagnostic and management algorithms to identify individuals at risk of MASLD in primary and community-based care, as well as referral pathways to ensure linkage to secondary and tertiary care.^121^^,^^124^

#### Coexisting MASLD and chronic hepatitis B

An additional challenge in low-resource settings is the growing burden of dual-aetiology liver disease, particularly the coexistence of chronic viral hepatitis and MASLD. Data from the European MASL-B registry (n = 1,709) and a global meta-analysis show that MASLD is present in over 36-40% of patients with chronic hepatitis B (CHB), and that disease severity and fibrosis progression are driven predominantly by host metabolic factors rather than viral activity.[125], [126], [127] Importantly, MASLD is independently associated with an almost three-fold increased risk of advanced fibrosis in CHB, whereas viral markers showed no independent association with fibrosis severity. This may be due to more viral persistence as comorbid MASLD significantly reduces intrahepatic interferon pathway activity, the expression of interferon-stimulated genes and macrophage gene signatures compared to CHB alone.^128^ Moreover, among patients receiving long-term antiviral therapy with effective viral suppression, MASLD – particularly insulin-treated T2DM – has emerged as the key determinant of advanced fibrosis, underscoring that in virally suppressed populations, metabolic liver disease rather than HBV *per se* appears to drive disease progression, as confirmed in the large SONIC–B study (n = 3,179). Importantly, in the latter study it was also observed that the presence of metabolic comorbidities in patients with CHB on antiviral therapy was associated with less fibrosis regression (adjusted OR 0.26 with ≥2 comorbidities) and a higher risk of fibrosis progression.^129^ Lastly, in a study of 1,089 patients with CHB and MASH from Europe and Canada, MASH was strongly associated with liver-related outcomes (liver decompensation, transplantation, HCC development) and all-cause mortality^130^ during 13 years follow-up. To assess HCC risk in those with CHB and MASLD, the PAGE-B score could also be safely applied, with negligible 5-year risks in those with low scores.^131^

These findings have important implications for low- and middle-income countries, where HBV remains endemic and access to antiviral therapy is expanding. There is no reason to expect the prevalence of MASLD in CHB to be lower in these settings; on the contrary, rapid urbanisation, nutritional transition, and rising rates of obesity and diabetes suggest that the burden of dual-aetiology liver disease is likely to increase further. Failure to recognise and address MASLD in patients with suppressed viral hepatitis risks underestimating fibrosis burden and missing opportunities for early intervention, particularly in health systems already strained by competing infectious and non-communicable disease priorities.

## Management at different stages of SLD

### Multidisciplinary approach

**Fig. 4:**
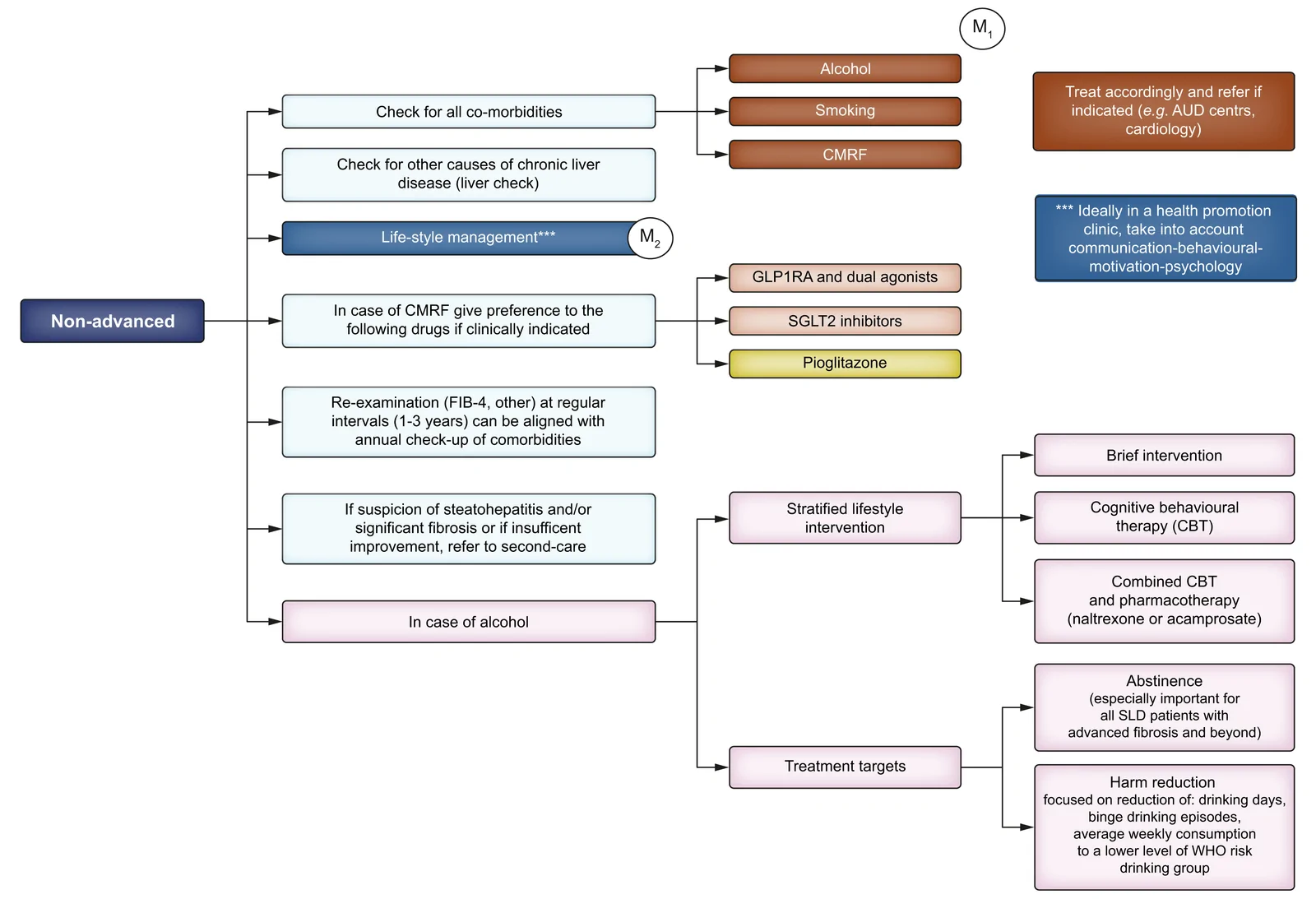
Management of people diagnosed with non-advanced SLD. AUD, alcohol use disorder; FIB-4, fibrosis-4; SLD, steatotic liver disease.

**Fig. 5:**
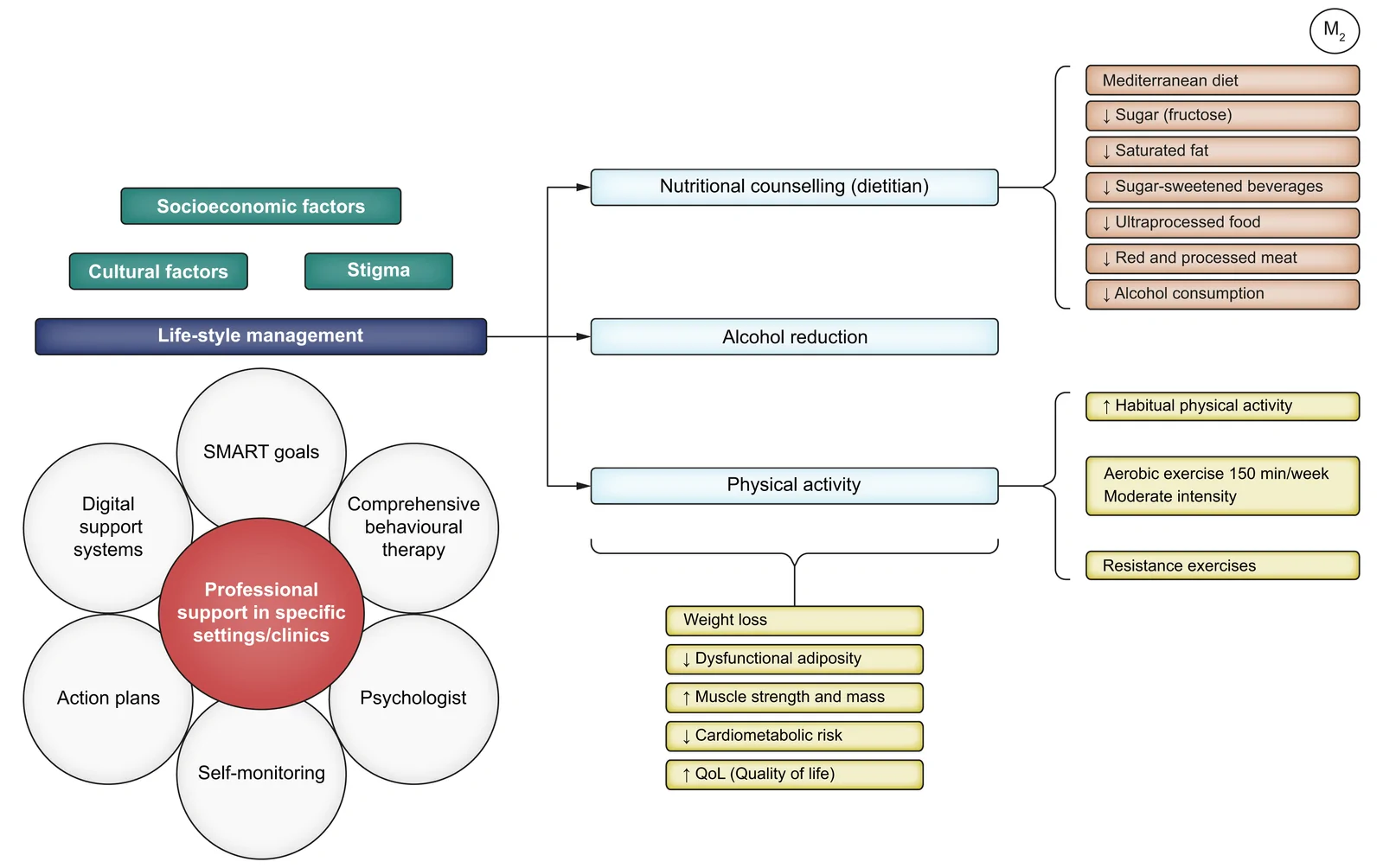
Lifestyle management of people diagnosed with non-advanced SLD. SMART, specific, measurable, achievable, relevant, and time-bound.

SLD is a multifaceted disease that requires individualised interventions based on the underlying driver of the disease, as well as the resources and socioeconomic circumstances of the patient. Ideally, it requires a multidisciplinary approach to tailor the care pathway to a specific patient. This is because SLD alone often reflects poor cardiometabolic health, of which a subset progresses to MASH and fibrosis, while the majority do not develop “at-risk” liver disease.^132^ A multidisciplinary approach should be used to address comorbidities and optimise referral pathways across different levels of care and resource availability, including nutritional, alcohol, and physical activity counselling. This multidisciplinary approach could be in part supported by digital application, digital therapeutics or teleconsultations. Currently, such pathways are not developed enough and are limited by the availability of resources and by reimbursement policies, resulting in inhomogeneous healthcare coverage. Insurance coverage is likely to evolve once specific MASH therapeutics become available; however, further action is needed among all stakeholders in the healthcare system to generate and integrate care pathways. Fig. 4, Fig. 5 summarise the management of SLD after identification of non-advanced disease.

**Fig. 6:**
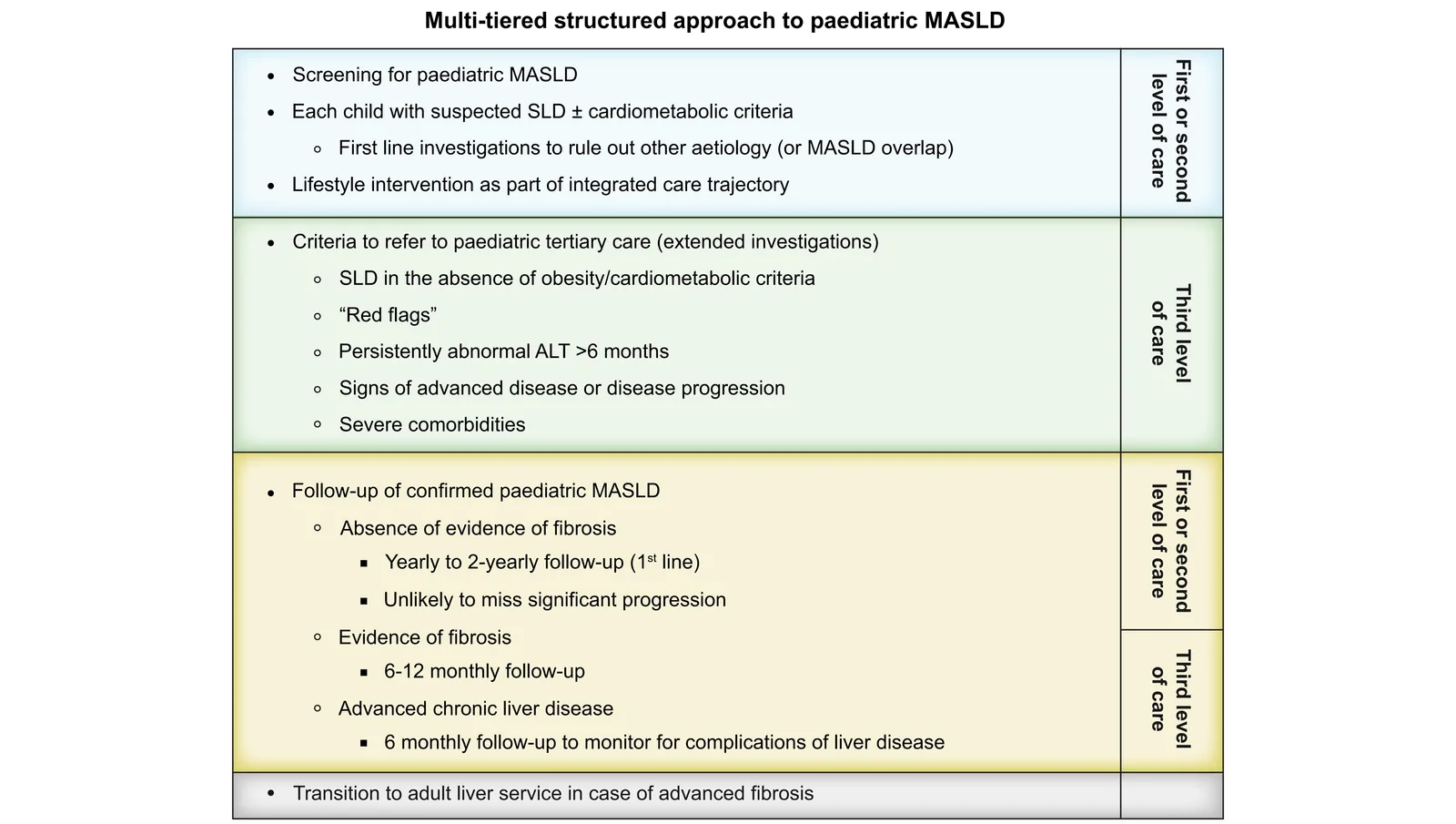
Multi-tiered structured approach to paediatric MASLD. ALT, alanine aminotransferase; MASLD, metabolic dysfunction-associated SLD; SLD, steatotic liver disease.

### How to approach SLD at different levels of healthcare: Primary care

#### Individualised healthcare recommendations

Primary care physicians are increasingly aware of liver disorders and specific guidance targeted to the management at the primary care level is required^1^. Primary care physicians can play a role at every stage of liver disease by increasing awareness, modifying risk factors such as alcohol consumption, smoking, obesity and eating habits and treating comorbidities.^6^^,^^96^^,^^133^ The development of health promotion centres (HPCs) (such as those in Slovenia)^134^ where eligible people are referred for behavioural lifestyle interventions by an interprofessional team of healthcare providers (including primary care physicians, nurses, psychologists, dietitians, physiotherapists/exercise specialists), is considered as a good practice.^135^ Smoking cessation support, alcohol control clinics or multidisciplinary primary care teams can be implemented when HPCs are not available. The effects of motivational interviewing and cognitive behavioural therapy in everyday primary care are apparent and there is strong support for using such techniques in the primary care setting.

For individuals diagnosed with SLD, primary care physicians play an essential role in diagnosing and managing common comorbidities such as T2DM, coronary heart disease, dyslipidaemia, obesity, and AUD, as well as in referring patients for further management in hepatology, obesity, or alcohol clinics, or to endocrinologists and other specialists. The role of polycystic ovary syndrome and obstructive sleep apnoea syndrome should not be underestimated, while kidney dysfunction should be investigated in all patients as an independent co-factor. In primary care, patients with SLD should be screened for the presence of significant fibrosis by NITs. Furthermore, in these individuals, all causes of SLD should be investigated. Individuals with incidental elevated transaminases (alanine aminotransferase (ALT) >33 U/L in males and >25 U/L in females)^136^ should receive an evaluation for SLD and for the other causes of chronic liver disease. Table 2 provides a suggestion for evaluation and further interventions.

**Table 2:** Assessment of SLD comorbidities in primary care and indications for referral.

SLD and comorbidities	Primary care investigation	Referral
Overall assessment of health status and intervention in overweight/obesity, alcohol, smoking, T2DM, dyslipidaemia, CHD (role of motivation and CBT). If unsuccessful intervention and/or signs of persistent or uncontrolled disease, patients should be referred at least once to a healthcare professional for an evaluation, according to current practice.		
T2DM	Fasting plasma glucose, HbA1c, oral glucose tolerance test, 2-h post-load glucose	Refer to diabetologist/endocrinologist for further investigation
CHD/-atherosclerosis	NT-proBNP (not available in all primary care settings)/CRP/blood pressure	Refer to cardiologist for cardiac ultrasound/investigation for heart failure
Dyslipidaemia	Triglycerides, cholesterol (total, LDL, HDL), lipoprotein (a) at least once and lipoprotein (b)	
Obesity	Body mass index Waist circumference Waist to height ratio Body roundness index TSH	Referral to HPC if available Refer to obesity specialist/endocrinologist, from there optionally to bariatric surgery clinics Referral to multidisciplinary weight management services if available
Kidney disease	Plasma and urine creatinine/serum and urine albumin Estimated glomerular filtration rate Cystatin C	Referral to specialist if renal failure is diagnosed Modifications to medications according to stage and treatment with new medications such as SGLT2 inhibitors
Obstructive sleep apnoea syndrome	Screening with STOP BANG questionnaire for example	Referral to sleep clinics for further investigation (if scores are eligible)
Polycystic ovary syndrome	LH, FSH, estradiol, testosterone, androstenedione, 17-OH-progesterone, DHEAS, SHBG	Referral to gynaecologist/endocrinologist
Hypothyroidism	TSH, FT4	Referral to endocrinologist
AUD	GGT, AST>ALT, MCV, CTD, AUDIT, AUDIT-C, PEth	Referral to AUD unit

ALT, alanine transaminase; AST, aspartate aminotransferase; AUD, alcohol use disorder; AUDIT, alcohol use disorders identification test; AUDIT-C, alcohol use disorders identification test–consumption; CBT, cognitive behavioural therapy; CHD, coronary heart disease; CRP, C-reactive protein; CTD, carbohydrate-deficient transferrin; DHEAS, dehydroepiandrosterone sulfate; FSH, follicle-stimulating hormone; FT4, free thyroxine 4; GGT, gamma-glutamyl transferase; GL, guidelines; HDL, high-density lipoprotein; HPC, health promotion council; LDL, low-density lipoprotein; LH, luteinizing hormone; MCV, mean corpuscular volume; NT-proBNP, N-terminal pro B-type natriuretic peptide; SLD, steatotic liver disease; SHBG, sex hormone binding globulin; SGLT2 inhibitors, sodium-glucose co-transporter-2 inhibitors; STOP-BANG questionnaire, snoring, tired, observed, pressure, body mass index, age, neck, gender; T2DM, type 2 diabetes mellitus; TG, triglycerides; TSH, thyroid-stimulating hormone.

#### Referral patterns

Individuals with mild or moderate risk could remain in primary care and should be assessed every 1-3 years with a NIT (*e.g.* repeat FIB-4 or other markers when further validated).^6^ All patients should be supported in achieving weight reduction, as well as alcohol and smoking cessation, in primary care and referred to specialised services if needed, as mentioned above. For individuals with a body mass index (BMI) >35 kg/m^2^, the need for specialised care and/or bariatric surgery should be discussed, and primary care physicians should refer patients to appropriate specialists for further evaluation. It is recommended that individuals with FIB-4 ≥1.3 and/or abnormal elastography findings (*e.g.* LSM ≥8 kPa by VCTE) should be referred to a hepatologist for further analysis of liver disease stage and aetiology. After the initial evaluation, if the patient is deemed at low risk of developing clinical liver disease, follow-up can be carried out by a primary care physician. Some individuals with LSM >8 kPa (F2) may need medical therapy and should be further managed in collaboration between the primary care physician and the hepatologist. In case of cACLD/cirrhosis (LSM >10 kPa, >F3) there is an increased risk for LREs. Follow-up includes screening for HCC with serum alpha-foetoprotein (AFP) and liver ultrasound every 6 months (which could also be considered on a case-by-case basis for those with F3), which is generally done in secondary or tertiary care. Dose adjustment and careful use of other medications, based on their safety profiles and the severity of liver disease, are essential.

#### Treatment options

Medications such as glucagon-like peptide-1 receptor agonists (GLP-1RAs) may be used in primary care for the treatment of individuals with MASLD/biopsy-proven MASLD with T2DM and/or overweight. Treatment options are discussed in more detail in the section on ‘antidiabetic and anti-obesity therapies’.

### How to approach SLD at different levels of healthcare: Metabolic health specialists (diabetologists, endocrinologists and obesity specialists)

#### Metabolic health specialists & liver health assessment

Diabetologists, endocrinologists and obesity specialists, collectively referred to as metabolic health specialists (MHS), are well positioned to manage non-advanced stages of SLD (MASLD and MetALD). Many individuals living with T2DM and/or obesity are also affected by SLD due to shared and/or mutually reinforcing pathophysiological pathways. In addition, T2DM and obesity are established risk factors for progression of MASLD to MASH with significant (F2) or advanced (F3–F4) fibrosis.^137^ As a result, the prevalence of individuals at intermediate or high risk of advanced fibrosis is higher in this clinical setting than in primary care.[138], [139], [140] There is a need to increase awareness of the importance of assessing liver health as part of the routine evaluation of comorbidities associated with T2DM and obesity, as underscored by the recent joint EASL/EASD/EASO clinical practice guideline.^6^

Importantly, the implementation of a systematic liver assessment will lead to the integration of liver status into the personalised management of these patients, along with other cardio-metabolic factors, such as CVD and chronic kidney disease.^98^ In this setting, the presence of liver fibrosis should be assessed using NITs, including LSM, or with liver biopsy at the discretion of the hepatologist.

#### Holistic management of SLD and associated comorbidities

Metabolic health specialists will take a holistic approach to SLD patient care, including lifestyle modification, optimisation of antidiabetic therapies, management of obesity and control of cardiovascular risk factors. Alcohol consumption should also be systematically evaluated. In case of harmful alcohol consumption (MetALD/ALD) or AUD, patients should be referred to hepatologists and/or other AUD specialists regardless of the severity of SLD. Otherwise, referral to hepatologists should be based on the severity of fibrosis, or to exclude other causes of chronic liver disease and SLD.

#### Referral patterns between MHS and hepatologists

Individuals with abnormal liver tests should always be evaluated for causes other than SLD, following the existing guidelines. Individuals with MASLD at low risk of liver fibrosis (VCTE <8 kPa) should not be referred to hepatologists and should be managed primarily by MHS. Lifestyle modification, including counselling on diet and physical activity, remains the cornerstone of management as described in more detail below in the section on structured lifestyle intervention for SLD. For some individuals, a psychologist specialising in eating disorders may be necessary. Such specialists are usually only available in secondary or tertiary centres for obesity management. Despite their importance, these resources are often underfunded and poorly reimbursed, which limits their accessibility. Therefore, shared platforms are needed to optimise patient care and should ideally be supported by public health policies.^5^ Individuals with MASLD at intermediate to high risk of significant fibrosis should be referred to hepatologists for further evaluation and management, as outlined in hepatologist section. Specific management strategies for patients with cirrhosis and cACLD are detailed in the relevant section.

#### Antidiabetic and anti-obesity therapies and liver health

Optimisation of antidiabetic and anti-obesity therapy in individuals with SLD should prioritise drugs with beneficial hepatic effects. GLP1-RAs or dual GIP/GLP1-RAs are preferred as they are associated with a significant improvement in steatosis, MASH resolution and fibrosis improvement (the latter demonstrated for semaglutide, with other trials ongoing).^6^^,^^141^ Alternatively, sodium-glucose transporter 2 (SGLT2) inhibitors are mainly associated with a reduction in liver fat content, although retrospective case-control studies also suggest a lower risk of LREs in patients with T2DM treated with SGLT2 inhibitors.^142^ Pioglitazone can also be considered in a select group of patients, but potential side effects such as weight gain, oedema and bone loss need to be considered, and it should not be used for patients with cACLD. Therefore, pioglitazone is currently not recommended for all individuals with MASLD.^6^ However, lower doses in combination with GLP-1RAs are effective and associated with fewer side effects.^143^

Intensive management of obesity should be considered, starting with lifestyle modification, followed by anti-obesity therapy with GLP-1RAs or dual GIP/GLP-1RAs or bariatric surgery, depending on patient preferences and risk assessment. Indeed, both strategies have demonstrated efficacy in improving liver histology in individuals with MASLD, although no anti-obesity medication is currently FDA-approved specifically for the treatment of MASH or liver fibrosis. Semaglutide at a higher dose (2.4 mg) led to resolution of steatohepatitis without worsening of fibrosis in 62.9%, and a reduction of liver fibrosis without worsening of steatohepatitis in 36.8% in a recent phase III trial of individuals with F2/F3 fibrosis and MASH and was granted accelerated approval by the FDA. Further, phase II trials of tirzepatide, survodutide and retatrutide also showed significant improvements in steatosis and possibly steatohepatitis.^141^^,^[144], [145], [146] Specific considerations in individuals with cirrhosis are discussed in detail in the section on surgery in MASLD-related cACLD.^6^^,^^141^

#### CVD risk management therapies and liver health

CVD risk management is warranted in individuals with MASLD given their doubled risk of atherosclerotic CVD (ASCVD) events,^147^^,^^148^ and the strong co-occurrence of cardiometabolic risk factors in both MASLD and ALD. Statins lower atherogenic low-density lipoprotein cholesterol by inhibiting cholesterol synthesis via HMG coA reductase in hepatocytes. Statins are effective in lowering ASCVD risk,^149^ yet are up to 33-50%Klik of tik om tekst in te voeren. under prescribed in individuals with SLD.^150^ This may be due to the misconception that they cause hepatotoxicity based on transient elevation of transaminases. Yet, a meta-analysis in 1,247,503 individuals^151^ indicated that statins are safe in MASLD, and even in compensated cirrhosis. Moreover, statin use was associated with lower histological steatosis grade and MASH activity, but not with less fibrosis. Statin use was also associated with a lower incidence of MASLD. In a cohort of 6,500 individuals with T2DM,^152^ statin use was associated with a reduced risk of HCC. A multidimensional study also found that statins ameliorate MASLD and lead to a greater reduction in ASCVD events in affected individuals than in those without SLD. Proposed mechanisms for the antisteatotic effects of statins are reduced lipid droplet accumulation via *SREBP1*, *PNPLA3* and *TM6SF2*,^153^ and inhibition of isoprenoid synthesis, as well as their impact on endothelial function.

#### Antiplatelet therapies and liver health

The role of platelets, as carriers of multiple mediators, as well as the role of altered platelet-endothelial interactions, is increasingly recognised as pathophysiologically relevant in cACLD in general and in MASLD in particular.^154^ Antiplatelet therapy, prescribed for secondary prevention of ASCVD events, may also exert beneficial effects in MASLD, potentially by limiting macrovesicular steatosis and intrahepatic immune cell trafficking.^154^ In a prospective study of 361 individuals,^155^ aspirin was independently associated with less severe histological MASH and less progression to advanced fibrosis. In a 6-month trial in 80 individuals with MASLD, daily low-dose aspirin significantly reduced steatosis compared to placebo.^156^

#### Referral patterns between MHS and general practitioners

Once metabolic comorbidities are managed and personalised intervention and therapeutic optimisation are established, patients should be referred to the primary care physician for follow-up. However, repeated liver assessment should be performed after 1 to 3 years, depending on the initial severity assessed by NITs, including LSM.^6^

### How to approach SLD at different levels of healthcare: Hepatologist

#### Liver disease: Confirmation of SLD diagnosis and stage of fibrosis

After an initial evaluation and decision on referral in primary care based on the FIB-4 score (or other established NIT in the future), secondary care by hepatologists should focus on further liver fibrosis risk stratification using more advanced NITs, as discussed in the sections on NITs in secondary care and tertiary care. Additionally, the hepatologist should strive to confirm or reject the diagnosis of SLD or other aetiologies through a thorough lab assessment, liver imaging and when needed liver biopsy, according to the clinical practice guidelines.^6^

Risk stratification should aim to assess the risk of outcomes and help guide treatment recommendations. Although there is currently no single NIT to exactly stage fibrosis in parallel to histological stages, surrogate markers of liver stiffness or collagen derivatives have shown high diagnostic accuracy^157^ to rule in or rule out the condition, and provide prognostic information.

#### Referral patterns between hepatologists, MHS and general practitioners

Individuals with concomitant insulin resistance or T2DM and/or alcohol use may be at higher risk of liver disease progression. ASCVD remains a major risk factor for mortality across SLD. Therefore, secondary care hepatologists should assess the individual ASCVD risk profile based on metabolic comorbidities and alcohol consumption when considering referral to the appropriate MHS. If patients are confirmed to have a low probability of advanced fibrosis (*e.g*. by either VCTE and/or or ELF), further management at a primary care level is possible with specific recommendations for lifestyle modifications and treatment of metabolic risks.^6^ Re-evaluation every 1-3 years is advised. Meanwhile, individuals identified with advanced fibrosis may be evaluated for MASH-directed pharmacotherapy if available (*e.g*. resmetirom or semaglutide), or referral to tertiary care for clinical trial evaluation and further assessment, including work-up for CSPH. However, implementation of referral pathways for individuals with SLD remains challenging due to the lack of reimbursement for secondary NITs in most countries, which reduces accessibility for many patients. Therefore, referrals are not as efficiently used or they are mostly focused on tertiary care (with rare exceptions like the United Kingdom, where the ELF test is reimbursed).

#### Specific MASH-targeted treatment

As noted, several drugs approved for other indications may have beneficial effects in MASH, with some showing histological evidence of MASH resolution and/or fibrosis regression. However, in the absence of phase III data and formal approval, they cannot be recommended specifically for MASH. Nonetheless, within their approved indications, their use may be considered in patients with MASH and fibrosis or non-invasive evidence thereof.^2^

Resmetirom^158^^,^^159^ has been approved by the FDA for MASH with significant or advanced fibrosis in the absence of cirrhosis and is in use in the US. Marketing authorisation by the EMA was granted in May 2025. The use of non-invasive biomarkers to initiate novel treatments and assess treatment responses is encouraging.^160^ Further research is needed, and reimbursement criteria will vary across healthcare systems.

Phase III data for semaglutide 2.4 mg in non-cirrhotic MASH^145^ led to accelerated FDA approval, and with several drugs in the pipeline, it is likely that the armamentarium of liver-directed MASH therapies will substantially expand soon. However, several issues remain unresolved, including non-histological diagnosis of treatment indication, assessment of treatment response, treatment duration, and treatment discontinuation.

### How to approach SLD at different levels of healthcare: The paediatrician

#### Diagnosis of MASLD in children

The diagnosis of MASLD in children and adolescents remains challenging due to the lack of accurate non-invasive diagnostic tools. According to the new nomenclature, paediatric MASLD can be diagnosed in the presence of hepatic steatosis in combination with at least 1 out of 5 paediatric cardiometabolic criteria (Box 2).^161^

Box 2 **Paediatric cardiometabolic criteria. Adapted from.161**

**Paediatric criteria: At least 1 out of 5**:

• BMI ≥85th percentile for age/sex [BMI z-score ≥+1] ***OR*** WC >95th percentile (***OR*** ethnicity adjusted equivalent)
• Fasting serum glucose ≥5.6 mmol/L [≥100 mg/dl] ***OR*** random serum glucose ≥11.1 mmol/L [≥200 mg/dl] ***OR*** 2-hour post-load glucose levels ≥7.8 mmol [140 mg/dl] ***OR*** HbA1c ≥5.7% [39 mmol/L] ***OR*** already diagnosed/treated T2DM ***OR*** treatment for T2DM
• Blood pressure: age <13 years old, BP ≥95th percentile ***OR*** ≥130/80 mmHg (whichever is lower); age ≥13 years old, 130/85 mmHg ***OR*** specific antihypertensive drug treatment
• Plasma triglycerides: age <10 years old, ≥1.15 mmol/L [≥100 mg/dl]; age ≥10 years old, ≥1.70 mmol/L [≥150 mg/dl] ***OR*** lipid-lowering treatment
• Plasma HDL-cholesterol ≤1.0 mmol/L [≤40 mg/dl] ***OR*** lipid-lowering treatment

BMI, body mass index; HDL, high-density lipoprotein; T2DM, type 2 diabetes; WC, waist circumference.

Alt-text: Box 2

#### Diagnostic work-up

In children with SLD, with or without concomitant abnormal liver blood tests, parallel diagnostic investigations remain essential to exclude alternative aetiologies, including in the presence of features of the MetS, to ensure that treatable diagnoses are not missed in children with overweight and obesity.^162^ Firstly, this includes testing for autoimmune liver disease, Wilson’s disease, alpha-1-antitrypsine deficiency, viral hepatitis and coeliac disease, as well as taking into account potential drug-induced liver disease and screening for alcohol use in adolescents. In the absence of abnormalities in the first-line investigations, clinical signs of chronic liver disease or severe elevation of transaminases (defined as ALT ≥80 IU/L), a tailored lifestyle intervention combining dietary change and physical activity can be initiated with re-evaluation after 6 months. In all other cases, or when abnormal transaminases persist after 6 months, a more comprehensive work-up is indicated, which should occur in the hands of paediatricians familiar with the management of paediatric liver disease (consisting of genetic testing, testing for inborn errors of metabolism affecting the liver, liver biopsy, *etc*., depending on the clinical context).^163^

#### Red flags: Referral to paediatrician

Specific “red flags”, warranting prompt referral, are young age (<8 years), lean children (BMI z-score <+1), growth failure, developmental delay, splenomegaly, synthetic hepatic dysfunction or signs of chronic liver disease, positive family history of liver disease and associated multisystemic disease or syndromic features.^163^^,^^164^ For each 1-unit gain in BMI-z score among children aged 7 to 13 years, the risk of cirrhosis increases by 16% in adulthood, and MASLD is an increasingly common cause of liver transplantation in young adulthood due to its high prevalence. Advanced MASLD increases the risk of non-liver-related complications, particularly T2DM and CVD.

#### Management of paediatric MASLD

MASLD and fibrosis might be more easily reversible in children.^165^ This emphasises the importance of early detection and early intervention to achieve sustained lifestyle modifications, which remain the cornerstone of treatment in paediatric MASLD. The tackling of sedentary behaviour and the reduction of fructose intake (via sugar-sweetened beverages including energy/power drinks) deserves emphasis in this population, particularly as adolescents have the highest intake of added sugars relative to other age groups.^166^^,^^167^ No pharmacological therapies are currently licensed in children living with fibrotic MASLD. Medications targeting weight loss (GLP-1RAs), now used in adolescents, may in the future be considered as an adjunct to lifestyle interventions when these are ineffective. However, the effect on paediatric MASLD is currently unknown.

#### Role of multidisciplinary teams and call for national policies

National policies should promote multitiered structured approaches (Fig. 5) to address childhood obesity and its comorbidities through prevention, early intervention and multidisciplinary management. Children and adolescents with moderate to severe MASLD should be managed by specialised multidisciplinary teams (including a paediatrician, a dietician, an exercise specialist, a psychologist and a social worker). In young children, family-based behavioural approaches are recommended, as parental involvement is crucial. Many adolescents with MASLD struggle with obesity, self-esteem or body image issues. Substance abuse and particularly alcohol consumption present another challenge in this age group, and adolescents should be counselled on alcohol use and its synergistic relationship with obesity. Liver biopsy should be considered in young people if advanced fibrosis cannot be confidently excluded. This should ideally be undertaken before transition to adult services to facilitate long-term care planning. During adolescence and young adulthood, patients are at increased risk of relapse and disruption of care, underlining the need for a structured approach to the transition from paediatric to adult MASLD care.^168^

### Structured lifestyle intervention for SLD (Fig. 6)

#### Nutrition in SLD

Lifestyle change is the cornerstone of treatment for SLD and should be adapted to individual circumstances (including, but not limited to comorbidities, alcohol intake, *etc*.) and sustainable in the long term, regardless of pharmacological therapies. “Nutritional counselling should be provided to all individuals with SLD, irrespective of disease severity, as it is important for preventing disease progression, improving liver damage, and reducing the risk of liver cancer and CVD.”.^6^ If resources are limited, individuals with significant or advanced fibrosis and/or multimorbidity (especially T2DM) can be prioritised. Structured lifestyle interventions should be readily available, accessible and affordable and should be widely recommended to individuals with SLD by HCPs across primary care, hepatology and other specialty clinics. Lifestyle interventions should be sensitive to stigma, economic constraints, and cultural variability. The work of HCPs should be based on an interprofessional approach that emphasises the coordination of treatment, integration of services and sharing of knowledge/expertise.^17^

Weight reduction by caloric restriction is key to improving MASLD biomarkers, including liver enzymes, liver steatosis, MASH, and fibrosis, either with or without increased physical activity.^169^^,^^170^ The exact caloric deficit should be tailored to individual needs and integrated into nutritional counselling. In adults living with MASLD and overweight/obesity, dietary and behavioural therapy-induced weight loss should aim for a sustained reduction of ≥5% to reduce liver lipid content, 7-10% to improve liver inflammation, and ≥10% to improve fibrosis.^6^ Normal-weight individuals with MASLD have a higher prevalence of metabolic alterations, including insulin resistance, greater visceral obesity, and decreased muscle mass, compared to normal-weight controls. They should aim to lose 3-5% of their weight, have a healthy diet and increase their physical activity levels following similar guidance to individuals living with overweight/obesity (see below and section on physical activity).^6^

Adherence to the Mediterranean dietary pattern prevents and treats MASLD,^169^ while unhealthy patterns increase the risk.^171^ The Mediterranean diet has the strongest evidence for reducing liver steatosis and confers benefits for cardiometabolic health.^172^^,^^173^ The Mediterranean diet advocates the reduction of sugars (including fructose) and refined carbohydrates, sugar-sweetened beverages, saturated fat, ultra-processed foods (typically rich in sugars and saturated fat), and red and processed meat, which are all related to increased MASLD risk in observational studies.[174], [175], [176], [177], [178], [179], [180], [181] Special emphasis should be placed on the restriction of sugar-sweetened beverages.[182], [183], [184] Examples of unprocessed/minimally processed foods recommended by the Mediterranean diet are vegetables, fruits (not juice), low-fat dairy, nuts, olive oil, legumes, unprocessed fish, and poultry. The principles of healthy eating recommended by the Mediterranean diet can also be applied in the form of low-carb diets or time-restricted eating,[185], [186], [187] and generally, long-term adherence can be improved by tailoring the dietary approach to the individual patient’s preferences, clinical, cultural, and socioeconomic characteristics.

Adherence to healthy eating patterns like the Mediterranean diet or similar healthy eating patterns may decrease the risk of developing HCC.[188], [189], [190] High intake of red meat, saturated fat, cholesterol, and refined sugars are associated with increased liver cancer risk.^189^ Specifically, sugar-sweetened beverages and red meat consumption (unprocessed and processed) were shown to be associated with an increased risk of liver cancer and liver disease-related mortality.^191^^,^^192^ Smoking is also related to MASLD, liver fibrosis and liver cancer.^193^ Furthermore, physical activity is related to a reduced risk of MASLD, HCC and liver-related mortality.^194^ Altogether, a healthy lifestyle is associated with a reduced risk of HCC.^195^^,^^196^

For effective implementation, HCPs should provide, in addition to giving general instructions for diet and physical activity, referral to comprehensive behavioural therapy, and help arrange regular follow-ups while emphasising to patients that this is a lifelong treatment. It is advised to use behaviour change techniques such as advising self-monitoring (*e.g*. food diary, step count), providing positive feedback on even small changes in lifestyle to enhance self-efficacy, explaining how lifestyle can significantly repair liver damage to increase motivation, setting realistic shared goals (SMART goals [Specific, Measurable, Achievable, Relevant, Time-bound]), and addressing barriers.^6^^,^^176^ Digital tools (including mobile apps, wearable devices, and online platforms) are still in the exploratory stage but seem promising.^197^^,^^198^ They can play a role in lifestyle implementation and self-monitoring in some patients, especially when there is difficulty in reaching regular in-person follow-ups,^197^ but they should not replace professional, personally tailored nutritional counselling.

#### Physical activity in SLD

People with SLD spend more time inactive than healthy controls,^199^^,^^200^ with each additional hour of sedentary time per day being associated with a 1.2% increase in liver lipid content.^201^

The vast majority (80%) of people with SLD do not meet the recommended physical activity guidelines and low levels of physical activity are associated with greater risk of liver disease progression and extrahepatic cancers.^202^ Generally, the more time spent being physically active, the greater the health benefits. However, the gains are especially significant for those currently doing the lowest levels of activity (<30 min per week), as the improvements in health per additional minute of physical activity are proportionately greater in this group.^203^ Increasing habitual physical activity throughout the day and reducing sitting time are important treatment targets for individuals with SLD – this helps to improve metabolic control and body composition, reduces the risk of developing CVD and T2DM, and burns more calories per day so is useful for patients who are trying to lose weight or maintain weight loss (see section on nutrition). Encouraging patients to look for opportunities to incorporate short periods of physical activity throughout the day is a useful strategy (*e.g.* walking up the stairs or parking further away from the shops), as cumulatively these small changes can make a difference.

Both aerobic and resistance exercise reduce liver lipid content in individuals with MASLD, independent of weight loss,^204^ with aerobic exercise offering the greatest benefit.^205^ Cardiorespiratory fitness (CRF) predicts mortality in MASLD (people with MASH have lower CRF than the general population).^202^ Therefore, it is important to incorporate structured aerobic exercise to improve CRF where feasible. Exercise also helps to prevent sarcopenia (resistance exercise is most effective; see sections on physical activity in cACLD) and improves overall quality of life. Guidelines recommend at least 150 min of moderate intensity aerobic exercise per week with the addition of resistance exercise.^6^^,^^205^^,^^206^ However, many of the individuals with SLD are a long way from meeting this target. Therefore, it is important to capture a baseline level of physical activity (*e.g*. using a physical activity calculator or average daily step count) for each patient and then provide tailored advice that considers current fitness, comorbidities, personal preferences, and socioeconomic circumstances in a bid to encourage individuals to gradually increase their activity and CRF levels. It is worth noting that although resistance exercise yields smaller changes in liver lipid content and CRF, resistance exercise has the lowest cardiorespiratory demand, so it may be more accessible to individuals with MASLD who are unable to undertake aerobic exercise.

The use of SMART goals, alongside action planning and self-monitoring, can help individuals to become more physically active.^207^^,^^208^ Activity trackers or pedometers can be useful tools to improve longer-term adherence by enabling patients to track their progress and by directing them to appropriate resources, both in the local community and online (including physical activity apps and online exercise programmes).^209^

#### Role of the nurse in SLD

Many patients face challenges in maintaining lifestyle changes due to multimorbidity and health inequalities, such as low socioeconomic status, low health literacy, and language barriers.^210^ This may result in suboptimal care and people with SLD report high rates of unmet physical and practical needs, especially with lifestyle changes.^211^ An interprofessional team is highly desirable and often essential to address this disease and its social complexities, with nurses playing a key role in clinical management, self-management support, and care coordination.

Nursing care in clinical practice is tailored to the nurse’s qualifications and to the disease stage.^212^^,^^213^ Nurses monitor surveillance protocols, assess liver function, and screen for complications. Advanced practice nurses, with their higher level of training, provide more autonomous care, including diagnostics, treatment planning, and prescribing medications. In early stages of SLD, nurses assess clinical variables (*e.g.* weight, liver stiffness), cardiovascular risk factors, comorbidities (*e.g*. AUD, frailty), psychosocial variables (*e.g*. quality of life, caregiver burden), and needs (*e.g.* physical, psychological, practical). In advanced stages, nurses can assist in monitoring disease progression, hepatic encephalopathy, falls, frailty, and complications like HCC and gastroesophageal varices.

Supporting self-management is a core nursing responsibility. Empowering patients and their informal caregivers to take an active role in managing the chronic condition improves health outcomes and shared decision-making.^214^^,^^215^ Nurses provide tailored education, emotional support, and foster behavioural changes for long-term health management. Educational topics include pathophysiology, symptom management, medication, and healthy lifestyle.^212^^,^^213^ Education depends on available counselling from other professionals (*e.g*. dietitians, physiotherapists, addiction specialists, and social workers). The delivery of complex behaviour change interventions begins with assessing readiness for change, followed by tailoring strategies to the patients’ needs to promote problem-solving and decision-making. While individual programmes have been shown to improve survival, reduce readmissions, enhance patient satisfaction, support alcohol reduction in cirrhosis, and improve quality of care, further research is needed to identify the key components of effective interventions.^215^^,^^216^ Digital health tools such as wearable devices, apps and more comprehensive digital health programmes enable remote monitoring (*e.g*. vital signs, hepatic encephalopathy, weight, fluid intake, well-being), support decision-making (*e.g*. guide symptom management, flag abnormalities, prompt timely interventions), facilitate education and behavioural changes (*e.g*. medication reminders, diet and physical activity guidance).^217^^,^^218^ These tools have the potential to provide efficient care, reduce readmissions, decrease the length of stay, and minimise unplanned paracentesis.

Nurses play a vital role in care coordination, often serving as the first point of contact. While nurse-led clinics may provide routine care,^219^^,^^220^ interprofessional teams are more effective in enhancing outcomes and care quality.^213^^,^^221^^,^^222^ As disease progresses, nurses manage increasing complexity by ensuring continuity of care, linking primary and hospital care, and fostering collaboration within interprofessional teams. Effective care coordination improves survival, and reduces healthcare utilisation and costs.^223^ As advocates in chronic disease management, nurses help patients navigate the healthcare system, connect them to resources, and facilitate specialist referrals. Particular attention should focus on: assisting patients with low health literacy in making sustainable lifestyle changes;^224^ supporting disadvantaged and marginalised patients who face barriers to high quality care;^210^ helping individuals with AUD access evidence-based treatment, as referral rates and participation remain suboptimal;^225^ and involving supportive and palliative care specialists early to enhance symptom control.^226^

### Motivation and information - a patient perspective

Starting a conversation about lifestyle change is important and some considerations need to be made. People living with obesity are nearly always aware of their excess body weight and know they need to change their lifestyle. They are usually aware of the health risks of being overweight, but rarely of the risks to the liver. They often feel ashamed, have started diets with good intentions and after a few months lapse into their old pattern in disappointment. Talking about this is difficult because they often already feel very guilty and often regard this failure as their own fault, without realising that several psychological, environmental and socioeconomic factors play a role (Table 3).^227^

**Table 3:** Recommendations on how to conduct a conversation about weight.

1. Ask permission to talk about weight
2. Ask about the history of being overweight and what attempts the patient has already made to lose weight and what difficulties he/she experienced
3. Ask if the patient is ready to make small changes and describe what kind of assistance you, as an HCP, can offer
4. Present a positive outlook for the future based on small adjustments
5. Discuss ways to change their dietary habits (including alcohol)/increase physical activity levels and let the patient choose something that he/she likes and that they think would be easy to adopt and maintain

Patient information can be found on the website of Liver Patients International.^227^

#### Unmet patient needs

Besides providing information on diagnosis, treatment options and the disease, HCPs should pay attention to “unmet patient needs”, used to describe conditions or symptoms that require additional attention but whose treatment or diagnosis are not addressed adequately by available guidelines or therapy. Unmet patient needs are personal and individual for each patient. Patients want an open, empathic dialogue and advice from their healthcare providers to be able to adapt to their disease and (new) lifestyle, treatment and care, and life. Examples of unmet needs are dealing with fatigue, digestive issues, pruritus, and pain, as well as impairments in their personal and professional lives, psychosocial issues in relation to their disease (anxiety, guilt), and the consequences of their health on their families’ lives.

### Flexible systems with different levels of approach, from the minimum basis to the ideal solution

Although SLD, and particularly MASLD, show a high prevalence globally, appropriate case finding and referral pathways for those with at-risk MASH and advanced fibrosis (F3), as outlined in practice guidelines, have not yet been widely adopted by primary care and specialties outside hepatology.^6^

As health systems move from *ad hoc* case finding toward proactive case finding for SLD among at-risk groups, the limitations of manual workflows and clinician-dependent heuristics become apparent. Tools developed for lower patient volumes do not readily scale to the demands of systematic risk stratification in primary care. Leveraging the ongoing digitalisation of healthcare – routine electronic health records (EHRs), laboratory information systems, and high clinician and patient adoption of digital platforms – offers a pragmatic pathway to deliver screening and early detection at scale with greater consistency and equity.

Automated clinical decision support can reduce cognitive load, standardise application of risk algorithms and cut-offs, and close knowledge gaps that often impede uptake in frontline settings. By automatically ingesting structured data already available (*e.g.* demographics, laboratory results, comorbidities, medications), digital tools can serve as real-time risk assessments and provide clear next step recommendations (*e.g.* repeat testing intervals, non-invasive fibrosis assessment, referral thresholds). Such automation is particularly valuable in primary care, where time constraints and variable familiarity with liver-specific pathways can hinder implementation. A concrete example is the LiverPRO score, a CE marked, medical device software tool currently being implemented in Denmark and Norway as a primary care solution. LiverPRO automatically captures relevant liver blood tests from laboratory information systems, generates a patient level fibrosis risk estimate, and returns actionable guidance to the clinician within routine workflows. By embedding the logic for appropriate cut-offs and downstream actions, systems like LiverPRO can improve adherence to evidence-based pathways, reduce unwarranted variation in referral and testing practices, and accelerate identification of patients most likely to benefit from further assessment. Beyond clinical efficiency, such tools may contribute to reducing health inequities by providing standardised, literacy independent guidance that does not rely on access to subspecialty expertise.

Digital solutions used by patients could help keep track of test results to guide follow-up examinations and deliver tailored treatment recommendations (*i.e*. lifestyle interventions). Wearables, including continuous glucose measurement and other handheld applications, may serve to provide immediate feedback on lifestyle choices. To increase retention in lifestyle interventions, peer-guided treatments or peer-to-peer programmes under the supervision of health coaches or specialty nurses can help support these changes. Access to individualised data or electronic healthcare records may increase the empowerment of patients to stay informed and monitor their own progress.

With the advent of pharmacotherapies, the implementation of referral pathways based on simple NITs may soon become more standard. While case-finding will largely shift to primary care, selection of patients for pharmacotherapy will rely on NITs such as LSM in secondary or tertiary care.^228^ More importantly, patients will have to be evaluated according to NIT cut-offs prior to initiation of treatment. Prescriptions will be made primarily by hepatologists since treatment response and liver-related adverse events need to be closely monitored.

## Cirrhosis and compensated advanced chronic liver disease

### Definition of cACLD

**Fig. 7:**
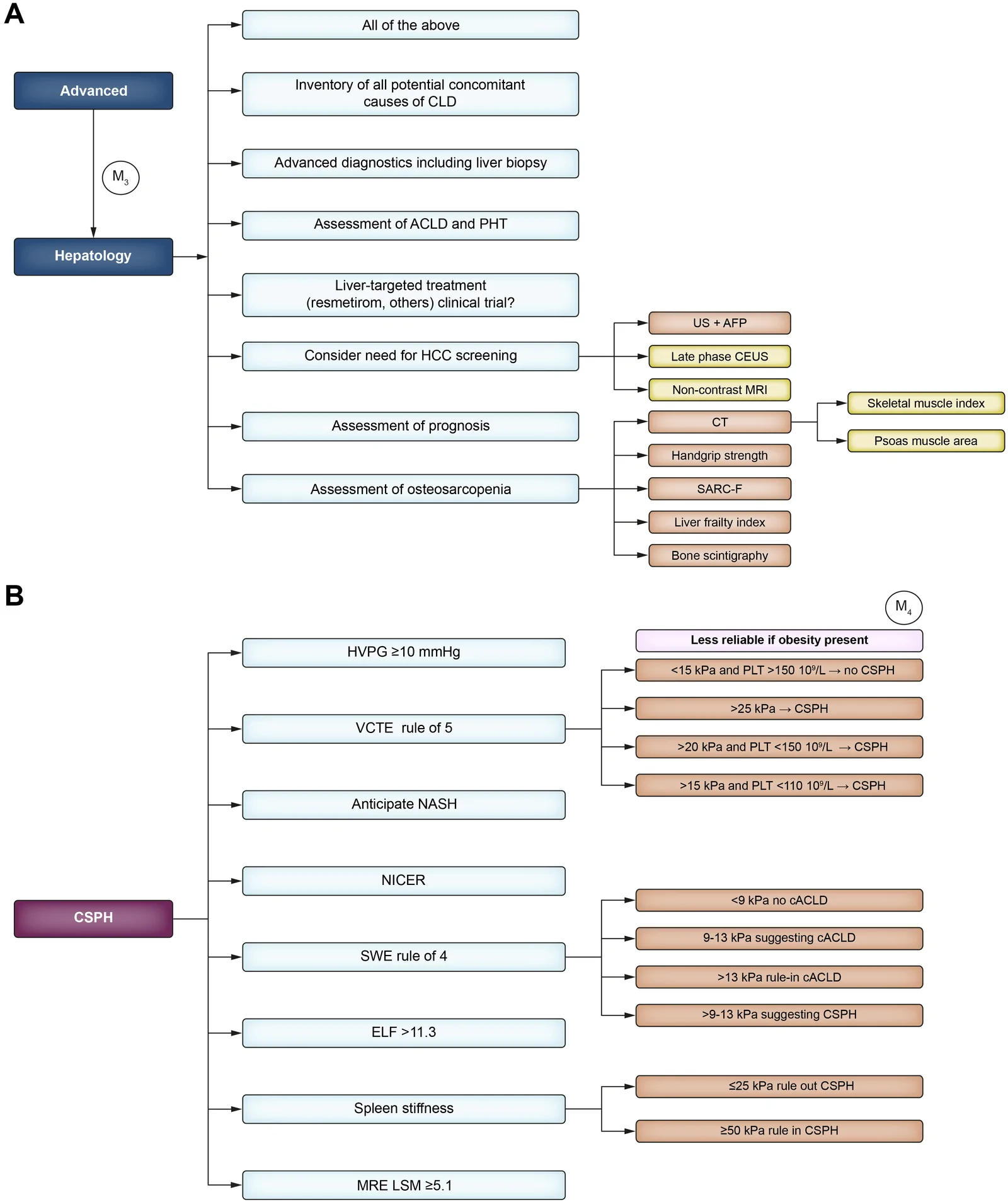
Management of people with advanced SLD, with or without CSPH. (A) Management of people with diagnosis of SLD with advanced disease. (B) Management of people with diagnosis of SLD with CSPH. ACLD, advanced chronic liver disease; AFP, alpha-fetoprotein; cACLD, compensated advanced chronic liver disease; CEUS, contrast-enhanced ultrasound; CLD, chronic liver disease; CSPH, clinically significant portal hypertension; CT, computed tomography; ELF, enhanced liver fibrosis; HCC, hepatocellular carcinoma; MRE, magnetic resonance elastography; MRI, magnetic resonance imaging; NICER, non-invasive CSPH estimated risk; PHT, portal hypertension; PLT, platelet count; SARC-F, strength, assistance walking, rise from a chair, climb stairs, and falls; SLD, steatotic liver disease; SWE, shear wave elastography; US, ultrasound.

The term compensated advanced chronic liver disease (cACLD) was originally proposed by the International Liver Pathology Group in 2012, to avoid the negative connotations of the term cirrhosis but also to capture the possibility of fibrosis regression using aetiological therapies. The Baveno VI consensus subsequently endorsed this definition “to better reflect that the spectrum of severe fibrosis and cirrhosis is a continuum in asymptomatic patients, and that distinguishing between the two is often not possible on clinical grounds”. Furthermore, in cACLD the risk of LREs and death increases significantly, and LREs become the dominant driver of outcomes (in contrast to CVD dominance in earlier stages). As in all the different stages of the disease, alcohol consumption and the presence of an AUD should be assessed (Fig. 7A).

### How to diagnose cACLD/cirrhosis – (non-)invasive tests

Liver biopsy remains the gold standard for the diagnosis of advanced fibrosis and cirrhosis, particularly when there are no overt signs of cirrhosis such as splenomegaly, low platelet count, venous collaterals or a nodular liver on imaging. The distinction between severe fibrosis with fibrous bridging and preserved liver architecture, and early cirrhosis with parenchymal nodules and fibrous septa, can be challenging to make using non-invasive fibrosis tests and is also dependent on the underlying liver disease. Some of the factors that could influence non-invasive testing include severe obesity, ascites, liver inflammation, and cholestasis.^229^

Liver biopsy is also useful if the cause(s) of liver disease is uncertain. Although ALD and MASLD share histological features in the early stages, several histological features, including occlusive lesions of hepatic outflow veins, extensive pericellular/sinusoidal fibrosis, excessive neutrophilic parenchymal inflammation, cholangitis, and cholestasis, implicate alcohol as a driver of chronic liver disease. The detection of alcohol as an aetiological factor is important because it is associated with worse prognosis, and it influences patient management.^6^ In cases with advanced ALD and decompensation, corticosteroids may be considered for the treatment of alcohol-related steatohepatitis. However, the accuracy of non-invasive methods for the diagnosis of alcohol-related steatohepatitis is not optimal.^230^ Liver biopsy may be required to confirm or exclude alcohol-related steatohepatitis on histological grounds. In addition, histological sub-staging of alcohol-related cirrhosis provides independent prognostic information.^231^ In cases with impairment of coagulation, liver biopsy may be performed via the transjugular route.^232^ However, the use of NITs has greatly reduced the need for a liver biopsy.

Simple NITs such as FIB-4 at a cut-off of <1.3 can rule out cACLD with a high negative predictive value. However, their use is not recommended in secondary care settings or for making a positive diagnosis, as their specificity is suboptimal. LSM with transient elastography is the best validated NIT for cACLD in secondary care; according to the Baveno VII criteria, values of <10 kPa and >15 kPa can be used to exclude and diagnose cACLD, respectively.^233^^,^^234^ Magnetic resonance elastography is slightly more accurate than transient elastography but less available and more expensive; therefore, it is not considered part of the standard of care.^235^

Importantly, liver stiffness provides prognostic information for LREs, which is integral to the concept of cACLD.^236^ Models that combine clinical parameters with liver stiffness, such as the ANTICIPATE-NASH model^237^ or the Agile 3+ score,^61^ have even better prognostic accuracy. Changes in LSM can provide further prognostic refinement. In a cohort of 2,508 individuals with cACLD, a 20% increase in LSM at any time was associated with an approximately 50% increased risk of hepatic decompensation and liver-related death.^238^ The exact magnitude of change corresponding with clinically relevant outcomes requiresfurther validation.

Of the serum tests, the ELF test is the best validated in individuals with SLD. Cut-offs of >9.8 and >11.3 predict LREs^239^ and can diagnose advanced fibrosis and cirrhosis, respectively.^240^ The use of concordant serum and imaging tests can increase the diagnostic accuracy for cACLD, particularly when the results are close to the cut-offs.^241^

### How to assess portal hypertension and clinically significant portal hypertension in MASLD cACLD/cirrhosis

CSPH is strongly associated with risk of decompensation, LREs and mortality in all chronic liver diseases, including MASLD (Fig. 7B). Indeed, in MASLD, the risk of hepatic decompensation and liver-related death is 5- and 15-fold higher, respectively, in those with CSPH.^242^ CSPH is diagnosed invasively by hepatic vein catheterisation measuring the hepatic venous pressure gradient (HVPG).^243^ However, HVPG is only available in expert centres, and therefore, the non-invasive assessment of the individual risk of having CSPH is extremely important. The Baveno VII “rule of five (kPa)” criteria identifies individuals with LSM <15 kPa by VCTE and normal platelet count (PLT) as not likely to have CSPH, while those individuals with LSM ≥25 kPa, LSM >20 kPa and PLT <150,000x 10^9^/L, or LSM >15 kPa and PLT <110,000 10^9^/L are considered at high risk of having CSPH.^244^ However, in individuals with obesity and cACLD related to MASLD, these criteria do not seem to apply, as the PPV decreases to only 62.8%.^234^ In fact, the higher the BMI, the greater the risk of misclassifying CSPH based on the original Baveno (ANTICPATE) criteria. Therefore, the ANTICIPATE-NASH criteria were developed to account for BMI; in this setting, the model has 90% power to discriminate CSPH[234], [80] and 80% power to predict 3-year LREs.^237^

The Baveno dual cut-off model leaves a certain grey-zone that could be diminished by adding another NIT to help the discrimination of non-classified individuals. Spleen stiffness measurement (SSM) </≥40 kPa assessed by a dedicated VCTE probe/protocol was shown to reduce the grey zone from 44% to 9%;^245^ similar reductions were observed in non-viral and obese individuals (from 31 to 5%, and from 35 to 6%, respectively).^245^ The ELF score could also be used as the second discriminator, values >11.3 having 51% sensitivity and 72% specificity for CSPH.^239^

In individuals living with obesity, the use of VCTE is challenging and frequently requires the dedicated XL probe for LSM (a specific probe for SSM in case of obesity is not yet available). As an alternative, SWE techniques could be used, with the additional benefit of enabling visual assessment of both the liver and spleen and other signs of portal hypertension. As the techniques are not similar, for SWE, it is recommended to use the rule of 4 (hence 5-9-13-17-21 kPa) with the same significance as the original Baveno endorsed rule of 5.^246^ When available, MRE could also be used to predict CSPH. In fact, AASLD recommends that all individuals with LSM between 15-25 kPa and PLT >150,000x 10^9^/L, as well as those with LSM <15 kPa and thrombocytopenia, should undergo MRE, with CSPH defined as LSM ≥5.1 kPa.^247^

Of note, hepatic decompensation in MASLD has also been observed with HVPG <10 mmHg.^248^ Although the CSPH threshold still confers a risk-stratifying value, the data suggest that HVPG measurement might underestimate the true portal pressure and hence portovenous pressure gradient.^242^^,^^249^ In this instance, spleen stiffness may represent a more reliable assessment of portal hypertension, but this warrants further study. Direct echoendoscopy-guided portal pressure measurement could be suggested in doubtful cases but is invasive and to date not extensively validated.

### HCC surveillance in MASLD cACLD/cirrhosis

Despite growing evidence of increased HCC occurrence in non-cirrhotic MASH (representing up to ∼30% of HCC cases), and the well-known association between obesity and diabetes and an increased risk of any type of cancer, including HCC (up to 4.5 times), HCC surveillance in non-cirrhotic MASH is currently not recommended.^250^ The precise incidence at different stages and cost-effectiveness considerations do not currently justify such a generalised surveillance approach. However, individuals with MASLD and advanced fibrosis and those with MASH would also benefit from a systematic HCC surveillance programme.^251^

The current concept in HCC is individualised surveillance, based on individual patient risk. This could be assessed by various scores, such as GALAD (consisting of gender, age, AFP, AFP-L3 and PIVKA-II), or aMAP (age, gender, bilirubin, albumin and platelets),^6^ or considering baseline liver stiffness.^252^

The rule of “ultrasound every 6 months” is challenged in individuals with MASLD and obesity, as they have an increased risk of inadequate ultrasound quality^234^ due to poor visualisation. Abbreviated non-contrast MRI^253^ or late phase contrast-enhanced ultrasound ^254^ could serve as alternative imaging screening methods, depending on local availability and experience. Bi-annual alpha-fetoprotein determination appears to be an adequate screening strategy in resource-limited settings or in individuals with poor ultrasound visualisation and no access to alternative imaging.^250^

### Prevention of liver decompensation and liver-related complications in MASLD-related cACLD/cirrhosis

Achieving effective, durable weight loss is of paramount importance in SLD. In cirrhosis lifestyle intervention can induce effective and durable weight loss^255^ and has a positive impact on portal hypertension.^256^ Long-term weight loss can also help to prevent progression to cirrhosis in individuals with advanced fibrosis.^257^ However, although caloric restriction is important for weight loss, risk of muscle wasting must be anticipated for individuals living with cirrhosis. Sarcopenia (a progressive decline in skeletal muscle mass and function) has been reported to be associated with increased morbidity and mortality, worse quality of life, and disability. Sarcopenia is associated with progression to liver failure and is a prognostic determinant in cirrhosis. Sarcopenia is also an independent predictor of mortality in cirrhosis and is associated with adverse outcomes, including portal hypertension, infections, HCC, hospitalisation, and worse outcomes after liver transplantation.^258^ Therefore, addressing and treating sarcopenia in cirrhosis has a major impact on survival.

#### Alcohol use and alcohol reduction in patients with cACLD and cirrhosis

Alcohol consumption is a major, modifiable driver of disease trajectory in cACLD and cirrhosis, regardless of underlying aetiology. Across the spectrum of liver disease, alcohol accelerates fibrosis progression, raises portal pressure, increases the risk of decompensation, and worsens survival. Risk is dose-dependent and begins at relatively low levels for vulnerable subgroups. Beyond epidemiology, haemodynamic data link alcohol exposure directly to portal hypertension. In alcohol-related cirrhosis, portal pressure and variceal size fall with sustained abstinence and rise rapidly after re-exposure. Crucially, high-quality outcome data demonstrate that abstinence changes prognosis at every stage of portal hypertension.^259^^,^^260^ In a multicentre cohort of alcohol-related cirrhosis with baseline HVPG ≥10 mmHg, abstinence independently reduced hepatic decompensation and liver-related and all-cause mortality – even among patients already at high risk of HVPG (≥20 mmHg). A 2024 meta-analysis pooling 19 studies (n = 18,833) found that abstinence was associated with ∼40% lower overall mortality (pooled HR 0.61, 95% CI 0.51–0.74) and a similarly lower risk of decompensation; the benefit held in both compensated and decompensated disease. Effects on incident HCC were inconclusive in cirrhosis cohorts, although older population-level analyses suggest HCC risk declines gradually with years of abstinence. Implementation in routine care should be systematic. Brief screening with the AUDIT-C should be repeated and combined with objective biomarkers when accuracy matters (*e.g*. phosphatidylethanol [PEth] in blood; ethyl glucuronide/ethyl sulfate in urine; carbohydrate deficient transferrin in serum) to improve detection of ongoing exposure. Embedding systematic screening, objective verification, and access to evidence-based AUD therapy into hepatology practice is therefore essential to improve survival and reduce decompensation in this population.

### Nutrition in MASLD cACLD/cirrhosis

**Fig. 8:**
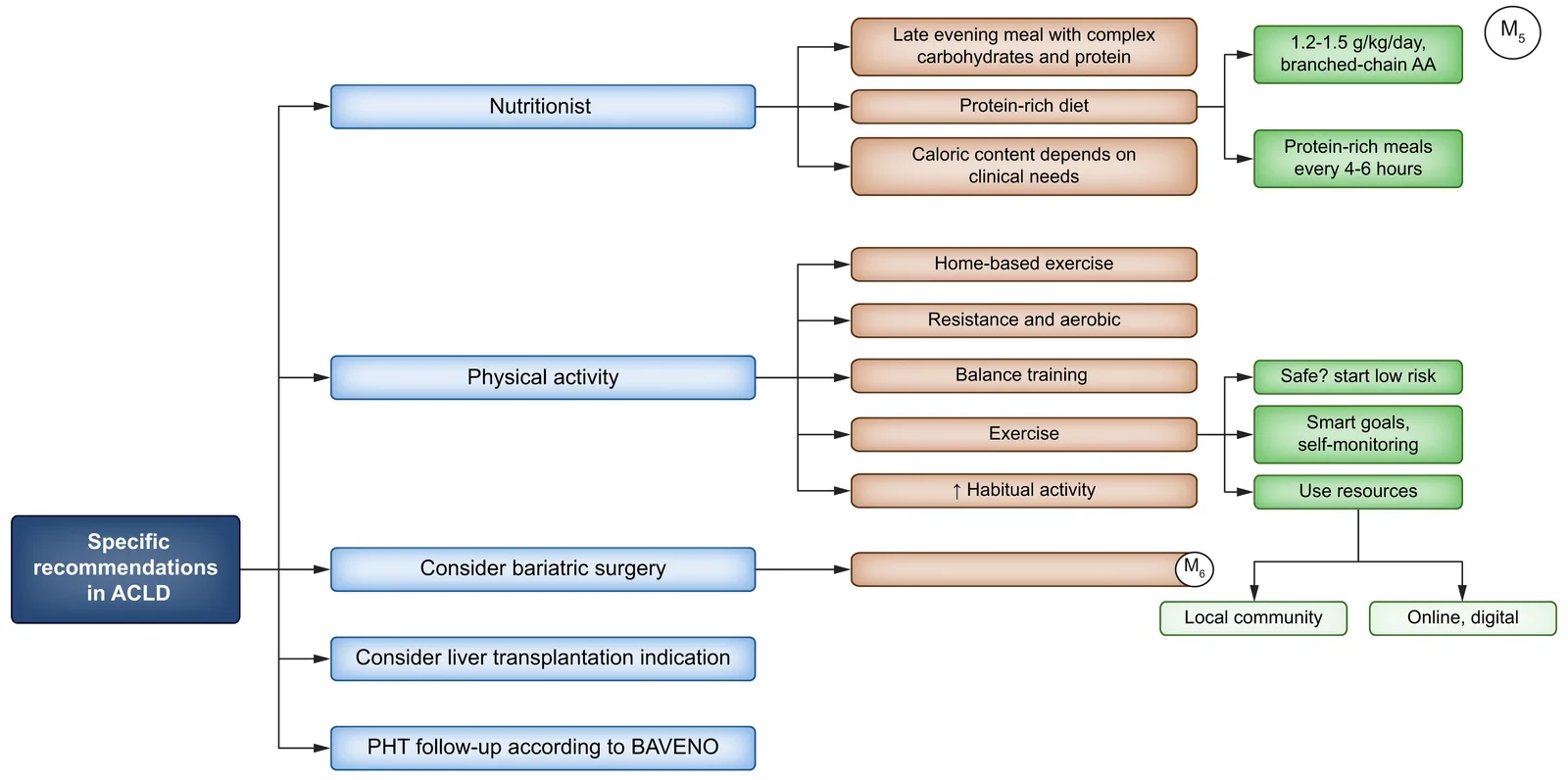
Management of people with SLD and ACLD. AA, amino acids; ACLD, advanced chronic liver disease; PHT, portal hypertension; SLD, steatotic liver disease.

Malnutrition and sarcopenia^261^ are highly prevalent in individuals with cirrhosis awaiting liver transplantation.^262^ Sarcopenic obesity is the state of decreased muscle mass together with increased adipose tissue mass.^263^ Sarcopenia can lead to frailty and a reduction in functional ability ^242^ and affects 22-62% of individuals with cirrhosis.^263^ Sarcopenia and frailty are predictors of poor liver-related outcomes and can negatively impact a patient’s quality of life (Fig. 8).^6^

Evaluation for sarcopenia includes the skeletal muscle index or psoas muscle area at the third lumbar vertebra via CT scan, and the measurement of frailty using tools like hand grip strength, SARK-F [FA1] or liver frailty index^6^ and other diagnostic procedures summarised in the joint ESPEN-EASO consensus statement.^111^

The EASL CPGs provide a comprehensive review and recommendations for the nutritional treatment of individuals with cirrhosis.^6^^,^^264^ Dietary and lifestyle recommendations should be adapted based on clinical parameters, including the severity of liver disease and its complications, comorbidities, nutritional status, the presence of sarcopenia or sarcopenic obesity, BMI, and age, as well as patient-specific factors such as symptoms (*e.g*. gastrointestinal, fatigue), appetite, and socio-economic barriers.

In adults with sarcopenia, sarcopenic obesity, or decompensated cirrhosis, it is recommended that a high-protein diet be followed, as well as eating a late-evening snack.^8^ High-calorie protein-rich diets improve nutritional status, sarcopenia, hepatic encephalopathy, survival, frailty, and quality of life in people with cirrhosis.[265], [266], [267] The recommended approach is to supply at least 35 kcal/kg of body weight/day, with a daily recommended protein intake of 1.2–1.5 g/kg of body weight/day (sufficiently rich in branched-chain amino acids) to prevent or reverse the loss of muscle mass.^6^^,^^261^^,^^262^^,^^264^ To prevent accelerated starvation and related proteolysis, there is a need to shorten fasting intervals between meals by eating every 4-6 h and having a late-evening snack.^264^ A late-evening snack containing complex carbohydrates and protein is beneficial.^268^^,^^269^

Moderate caloric restriction and weight reduction through lifestyle interventions can be suggested in adults living with compensated cirrhosis and overweight/obesity, with an emphasis on high protein intake and physical activity/exercise to maintain muscle mass and reduce the risk of sarcopenia^6^^,^^256^^,^^270^^,^^271^ (see section 6 on physical activity in cACLD).

### The importance of physical activity in MASLD-related cACLD

Physical inactivity can be a major cause of deterioration in individuals with cACLD/cirrhosis – lower daily step counts are associated with an increased risk of death or decompensation and the development of sarcopenia^209^ (Fig. 8). Targeting an increase in physical activity is essential in the management of sarcopenia in individuals with cACLD/cirrhosis and can lead to improvements in functional ability, muscle mass, and endurance, improving overall quality of life.[272], [273], [274] Small-scale studies of individuals with cirrhosis have shown that structured exercise is safe,[275], [276], [277] but the optimal exercise programme remains unknown. A combination of resistance and aerobic exercise may help to maintain/increase muscle mass and improve physical function.^205^^,^^272^^,^^274^^,^^278^ Home-based exercise has been demonstrated to be safe and well tolerated in individuals with cirrhosis,^272^^,^^273^^,^^278^ providing a low-cost, scalable option. An increase in day-to-day habitual physical activity should be encouraged alongside structured exercise to maintain independence; balance training should be incorporated for frailer patients to reduce risk of falls.^278^^,^^279^

Practically, it is important to ensure that individuals with cACLD/cirrhosis are safe to start an exercise programme. This involves considering liver disease-related safety issues (*e.g*. high-risk varices, high volume ascites), screening for cardiovascular safety, the impact of exercise on comorbidities (*e.g*. diabetes, musculoskeletal conditions) and the risk of falls due to frailty/sarcopenia.^279^ Advising patients to start at a low level, increase gradually and be alert for symptoms (both liver and non-liver-related) is key to improving the safety of an exercise programme.^279^ Chair-based exercises are often a useful starting point for patients with a low baseline level of fitness or at risk of falling. Incorporating counselling about the benefits of physical activity/exercise, establishing a baseline, and tailoring recommendations based on the patient’s day-to-day functional ability, comorbidities, personal preferences, and socioeconomic circumstances, should be a core part of routine care. Accompanying this with SMART goal setting, action planning and encouraging self-monitoring of activity levels can help to support patients in becoming more physically active.^205^^,^[207], [208], [209]^,^^279^ Additionally, signposting to appropriate resources, both in the local community and online, helps to foster longer-term adherence.^207^

It is essential that individuals with cACLD/cirrhosis are referred for dietetic counselling to assess and optimise nutritional status alongside initiation of a physical activity/exercise programme (see section on nutrition in cACLD). This helps to identify malnutrition and prevent exercise-induced muscle breakdown.^205^^,^^279^^,^^280^ Nutritional requirements will likely change as a result of increasing physical activity levels, with special emphasis on adequate protein intake, and it is important that this is reviewed regularly and adapted as necessary to optimise patient outcomes.^279^

### Surgery in MASLD-related cACLD

#### Bariatric surgery

**Fig. 9:**
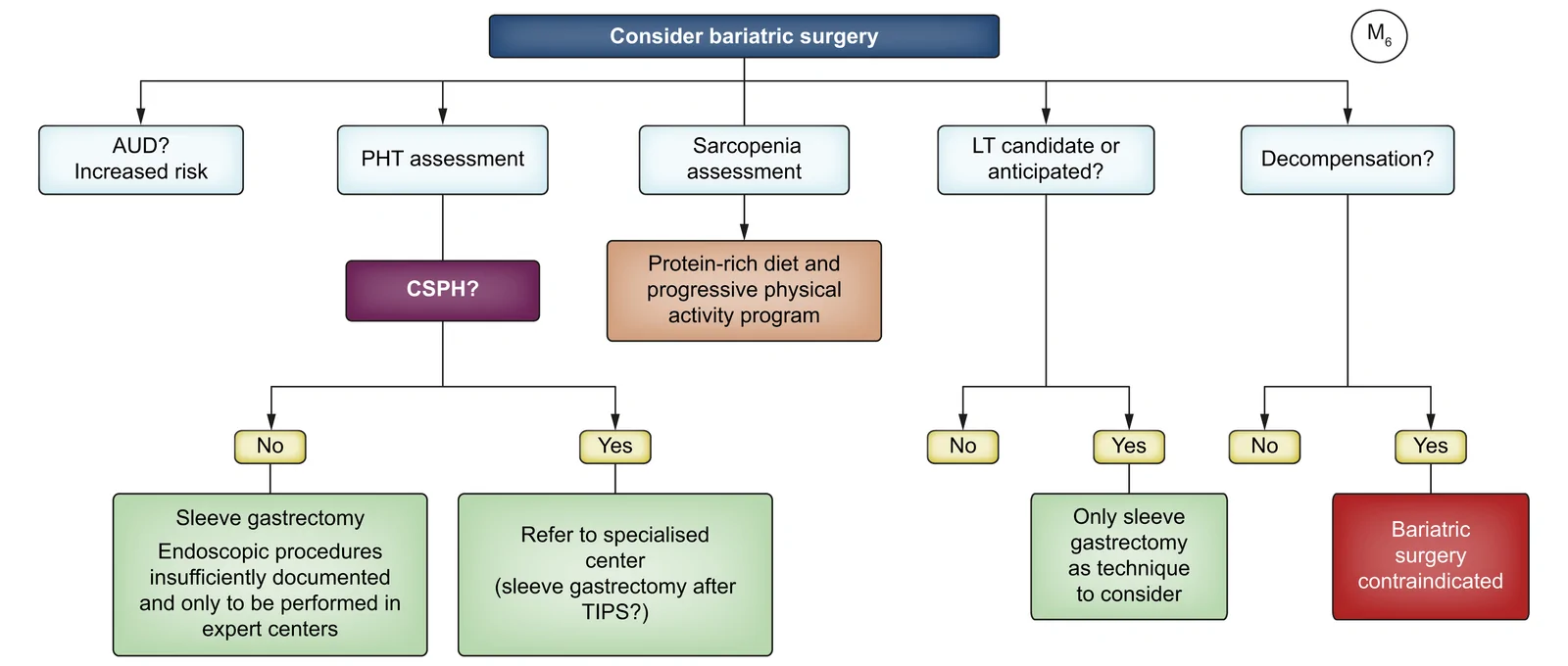
Bariatric surgery in people with SLD. AUD, alcohol use disorder; PHT, portal hypertension; LT, liver transplantation; SLD, steatotic liver disease; TIPS, transjugular intrahepatic portosystemic shunt.

Even at the stage of cirrhosis, bariatric surgery might offer benefit in carefully selected individuals with compensated cirrhosis (MELD score <12, Child-Pugh A), both in terms of cardiorenometabolic alterations and liver histology (Fig. 9). In decompensated cirrhosis, bariatric surgery is contraindicated because of a higher risk of complications and death^281^ (Fig. 9). Bariatric surgery is associated with high rates of MASH resolution (56–80% at 1 year); however, fibrosis improvement typically requires longer follow-up (up to 5 years) and may persist despite weight loss in many patients.^282^^,^^283^ Importantly, bariatric surgery studies performed specifically in individuals with compensated cirrhosis are lacking, and only a small proportion of individuals with advanced fibrosis or compensated cirrhosis have been included in available studies. In this subgroup, the proportion of individuals with fibrosis improvement is lower than in individuals with fibrosis stage F1–F2, ranging from 45.5% to 53%. Cirrhosis reversal has been shown to reduce the risk of liver decompensation,^267^ but whether this also applies to bariatric surgery-induced cirrhosis reversal (if it occurs), has not been proven. Given the impact of weight loss on portal hypertension,^268^ a reduced risk of related complications is to be expected regardless of cirrhosis reversal, but this has not been clearly documented in this patient group.^256^^,^^284^

Any candidate for bariatric surgery should be screened for MASLD and MASLD-related advanced fibrosis/cirrhosis. If cACLD/cirrhosis cannot be reliably ruled-in or -out, liver biopsy should be considered, ideally transjugularly, not only because of body composition and, if present, thrombocytopenia, but also as it allows for assessment of portal hypertension (and a right heart catheterisation can be performed in the same setting, assessing cardiac function). On top of the general considerations regarding bariatric surgery in MASLD, three elements require specific attention. First, the severity of portal hypertension should be accurately assessed, as described above. Ultrasound should be performed (or additional imaging in case of insufficient image quality) as well as an oesophagogastroduodenoscopy to check for signs of portal hypertension. HVPG measurement can be performed if available. Spleen stiffness^284^ needs further validation, but holds promise, because HVPG can underestimate the severity of portal hypertension in this population.^285^ In case of CSPH (documented or highly suspected), bariatric surgery should only be performed in high-volume centres that have specific expertise in both managing cACLD and bariatric surgery in cACLD/cirrhosis.^6^^,^^270^ Transjugular intrahepatic portosystemic shunt (TIPS) can be considered pre-surgery to reduce operative risks. Second, sarcopenia is an even greater issue in this patient group than in the overall patient population. Of particular concern is the potential worsening of sarcopenia associated with surgery-induced weight loss. Assessment of sarcopenia should be performed as described in section on nutrition in cACLD. A protein-rich diet and a physical activity programme for progressive muscle strengthening should be implemented pre-surgery and continued post-surgery and this should be supervised by appropriately trained HCPs. Third, although Roux-en-Y gastric bypass has been performed in these circumstances and is an option (again only to be performed in centres with specific expertise in bariatric surgery in cACLD/cirrhosis), sleeve gastrectomy is generally the recommended technique^6^^,^^270^ and also has the advantages of gradual weight loss, absence of malabsorption, and the preservation of endoscopic access to the biliary tree. However, sleeve gastrectomy appears to be less effective in improving fibrosis in individuals with advanced fibrosis.^283^

The question of whether the patient is a potential candidate for liver transplantation, now or in the future, should always be formulated, and if the answer is positive, sleeve gastrectomy is the technique of choice, given, amongst others, the impact of Roux-en-Y gastric bypass on absorption of immunosuppressive drugs. The optimal timing and type of bariatric surgery are also crucial. Bariatric surgery may be performed prior to liver transplantation with expected improvement on liver transplant candidacy and increased survival after transplantation. However, individuals who undergo bariatric surgery before liver transplantation must be closely monitored for nutritional status. For individuals with decompensated cirrhosis, the options are sleeve gastrectomy concurrent with or after transplant. Simultaneous transplantation and sleeve gastrectomy can be a safe option in expert centres, provided that the transplantation is a straightforward procedure.^286^

The safety and efficacy of endoscopic weight-loss procedures require further documentation in this particular patient population.^6^^,^^270^

An important aspect to consider is the effect of alcohol after bariatric surgery. Bariatric surgery has been linked to an increased risk of AUD, in particular after Roux-en-Y gastric bypass. The reasons for post-bariatric surgery AUD are multifactorial, including alterations in alcohol kinetics, which lead to higher peak alcohol concentrations and a greater feeling of drunkenness. The shift in addiction behaviour following bariatric surgery may lead patients to replace overeating with substance addiction, such as alcohol. The risk of AUD varies with the type of weight loss surgery and appears lower after restrictive procedures. The effect of prior bariatric surgery and alcohol use can lead to severe ALD at younger age.^287^ Bariatric surgery patients should therefore be educated about the possible risks of alcohol, and obesity management teams should be reluctant to perform bariatric surgery in individuals with a history of AUD.

#### General surgery, including cardiothoracic surgery and abdominal surgery

People with cirrhosis in general are at increased risk of complications and death when undergoing surgical procedures, but the risk is difficult to estimate and there are very few clear cut-offs that can guide decision-making. There is little data for MASLD-related cACLD/cirrhosis specifically, so the recommendations are general and not aetiology-specific. MELD,^288^ Child-Pugh score and Mayo Postoperative Mortality Risk (see EASL Guidelines App) are helpful for risk stratification.^289^

A general recommendation is that, besides carefully weighing the indication for the procedure, major surgical procedures in individuals with cirrhosis should be performed in high volume centres that combine expertise in the procedure with expertise in the management of individuals with cirrhosis and end-stage liver disease.

In case of liver resection, estimation of functional liver remnant volume (combining a volumetric assessment, *e.g*. MRI, with a functional assessment, *e.g.* hepatobiliary excretion scintigraphy) is required.

The severity of portal hypertension should be assessed (HVPG measurement, abdominal ultrasound, other abdominal imaging for collaterals, LSM, oesophagogastroduodenoscopy [spleen stiffness could be considered]) and CSPH is considered a contraindication for major abdominal surgery and is likely also an important risk factor in other types of surgery. TIPS could be considered to reduce the risk of portal hypertension-related complications and overcome CSPH as a contraindication, again only in centres with relevant expertise.

For hepatobiliary surgery, HVPG <10 mmHg, MELD score <9 and LSM by VCTE <22 kPa are recommended cut-offs.^290^^,^^291^ For major cardiac surgery, which is not infrequently needed in the MASLD population, a Child-Pugh score <8 or MELD score <13.5 is currently recommended.^292^^,^^293^ Alignment of risk stratification between all the different types of major surgery is desirable but there are currently no uniform criteria across all procedures. A comprehensive table can be found in.^289^

There is still a lot of misunderstanding about the bleeding risk in cirrhosis, as international normalised ratio does not reliably reflect the bleeding tendency in these patients. A platelet count >50,000x 10^9^/L (which can be achieved by the administration of thrombopoietin analogues in elective conditions and thrombocyte transfusion in acute circumstances), an international normalised ratio <2.5 and a plasma fibrinogen >100 mg/dl are generally considered safe and do not require further correction. More vigorous correction towards normal values may increase thromboembolic risk and exacerbate portal hypertension and should therefore be avoided. Viscoelastic testing (thromboelastography) provides a more accurate assessment of bleeding tendency and should guide perioperative coagulation management in major surgery in patients with cirrhosis.^294^

Careful consideration should be given to the use of medications, given altered hepatic metabolism (*e.g*. sedatives), increased risk of renal impairment (*e.g*. nonsteroidal anti-inflammatory drugs), and hepatotoxicity (*e.g*. acetaminophen)).

The presence of ascites, if not clinically apparent, should be assessed by ultrasound. Ascites is a sign of CSPH, may increase the risk of any surgery (*e.g*. because of respiratory consequences) and is a major decompensation event after surgery, in particular any abdominal or abdominal wall surgery. It is a relative contraindication to such a surgery, and treatment (which may include TIPS) should be considered. Ideally, depending on the indication and the urgency, surgery is only performed when ascites has completely resolved.^289^ Even then, the risk of recurrence is not negligible.

Child-Pugh C >10 or MELD >20 are generally proposed as cut-offs that contra-indicate major surgery.^273^ Liver transplantation should be considered, particularly if the estimated risk of mortality is >15% at 3 months or MELD >15.^288^

### Liver transplantation in MASLD cACLD/cirrhosis

In general, the indications and contraindications for liver transplantation (Child-Pugh B–C, MELD >15, HCC) are not different from other indications, but some aspects deserve consideration. Given the slow natural history of MASLD/MASH, patients tend to be older than those with other aetiologies of chronic liver disease at the time they pose an established indication for liver transplantation. This issue of age, together with the higher prevalence and severity of cardiorenometabolic comorbidities, often precludes liver transplantation in these patients. If they undergo liver transplantation, their outcomes are comparable to other aetiologies.^295^

Although much of this applies to any stage of MASLD, comorbidities should be thoroughly assessed in people with MASLD-related cACLD/cirrhosis and intensively managed according to the relevant guidelines. Systemic blood pressure, lipid profile and glycaemic control should be optimised.^296^ Although the presence of cirrhosis warrants caution in the use of certain drugs, most medications, including statins, aspirin and metformin, can be safely used in patients with Child-Pugh A cirrhosis.^283^

Sarcopenia is a particular concern. Muscle volume and strength need to be assessed in individuals with MASLD-related cACLD/cirrhosis and interventions (protein-rich diet, progressive exercise programme – see relevant sections) should be initiated in all patients, especially if liver transplantation is anticipated.

Although individuals with MASLD are at high risk of CVD and MASLD contributes to both atherogenic CVD as well as heart failure with preserved ejection fraction,^297^ it is often clinically less apparent in end-stage liver disease because of the haemodynamic changes induced by the liver condition (low systemic vascular resistance and low cardiac afterload). If CVD is only detected at the time of evaluation for liver transplantation, this can substantially delay transplantation (*e.g*. because of a period of non-interruptible antiaggregant/anticoagulant therapy after stent insertion, precluding major surgery) or even preclude transplant listing. It is therefore paramount to send individuals with MASLD-related cACLD/cirrhosis for a thorough cardiovascular assessment, at diagnosis and if evaluation for liver transplantation is anticipated. This should include cardiac ultrasound and effort testing/stress echocardiography,^298^ as well as other investigations, with a low threshold for testing at the discretion of the treating cardiologist. Ideally, this is performed by cardiologists who have experience with individuals with cACLD/cirrhosis and end-stage liver disease.

There should be a low threshold to refer patients to a liver transplant centre to evaluate potential indications for timely liver transplantation. During follow-up, even in the absence of a clear decompensating event, evidence of disease progression should prompt aggressive assessment of comorbidities and timely, in-depth investigation and management.

A high BMI, although complicating surgery technically and increasing the risk of post-operative complications, is no longer considered a contraindication for liver transplantation, but a BMI <40 kg/m^2^ is desirable.^299^ This can be achieved through any weight loss strategy but should be carefully guided and monitored, given the complexity of these patients’ clinical status, particularly dietary restrictions, and the substantial risk of worsening sarcopenia. As highlighted in the section on bariatric surgery, bariatric surgery is often contra-indicated at the time of transplant evaluation. The indication for and timing of bariatric surgery in this context should again be evaluated on a case-by-case basis by a multidisciplinary expert team. Sleeve gastrectomy at the time of transplantation,^300^ or in the post-transplant setting,^301^ can be considered. Roux-en-Y gastric bypass is not recommended because of the impact on the pharmacokinetics of immunosuppressives and other classes of drugs. Close surveillance of all comorbid conditions is warranted after liver transplantation to improve outcomes, which are mainly dictated by cardiorenal problems and may be exacerbated by MASLD recurrence.^302^

### Safety considerations for statins, antidiabetic and anti-obesity medications in MASLD-related cACLD

#### General considerations

In individuals with MASLD-related cACLD/cirrhosis, the use of medication can be challenging and safety considerations are warranted when managing common comorbidities including T2DM, dyslipidaemia and other cardiovascular risk factors. While physicians main concern is to avoid worsening liver function, the risks of precipitating renal failure, gastrointestinal bleeding, or hepatic encephalopathy are higher in individuals with cirrhosis.^303^

For example, non-steroidal anti-inflammatory drugs might precipitate both renal dysfunction and gastrointestinal bleeding and are thus strongly discouraged. However, paracetamol can be used safely when prescribed in relatively small doses (2-3 g or less/day) for short durations and is recommended as first-line treatment for pain.

Guidelines for over 200 different medications have been formulated, considering reported safety, additional risks and the need to change doses according to the stage of cirrhosis.^304^^,^^305^

A general rule should be “Where evidence is low lower doses are generally recommended” especially for higher degrees of liver impairment (Child-Pugh B, C).

Commonly used drugs that are contraindicated due to altered pharmacokinetics include several calcium antagonists (*i.e.* barnidipine, isradipine, nicardipine (PO), nitrendipine), and the beta blocker nebivolol.^303^^,^^304^

Very few drugs have been documented to have their hepatotoxic potential enhanced by cirrhosis; most involve antituberculosis agents or antiretroviral agents used for HIV or viral hepatitis. In cirrhosis, decreased therapeutic response is seen with beta adrenoreceptor antagonists, diuretics (furosemide, triamterene, bumetamide) and codeine. Proton pump inhibitors have been linked to an increased risk of spontaneous bacterial peritonitis, and dose adjustment is suggested, while lansoprazole and pantoprazole are considered unsafe. Cimetidine is also considered unsafe.

#### T2DM

When it comes to T2DM, which is highly prevalent in individuals with cirrhosis/cACLD, its management depends on the severity of cirrhosis and liver function status. For individuals with preserved liver function, glycaemic targets and therapies are similar to those in individuals without cirrhosis. In individuals with impaired liver function, individualised glycaemic control targets depend on life expectancy and potential side effects. Individuals with cirrhosis are at higher risk of hypoglycaemia due to chronic malnutrition, decreased glycogen stores, sarcopenia and associated chronic kidney disease.^306^ Thus, in individuals with severely impaired liver function (Child-Pugh C), only insulin therapy should be initiated.^307^ Whereas most antidiabetic therapies are safe in individuals with preserved liver function (Child-Pugh A), sulfonylureas and glinides should be used with caution and pioglitazone should be avoided due to the lack of evidence. SGLT2 inhibitors should be used with caution in individuals with chronic alcohol consumption due to the increased risk of ketoacidosis.^308^ In frail individuals, careful initiation and titration of GLP-1RAs is warranted because of the potential risk of dehydration and acute renal failure, due to gastrointestinal adverse events.^308^ No antidiabetic treatment has yet demonstrated efficacy in improving histology or reducing LREs in randomised controlled trials involving individuals with cirrhosis.^308^ Metformin has been associated with a lower risk of LREs, including hepatic decompensation, HCC, transplantation, and death, in individuals with T2DM and Child–Pugh A cirrhosis.^309^ A similar study reported a lower risk of adverse liver outcomes in individuals with MASH cirrhosis treated with GLP-1RAs compared to other antidiabetic therapies.^310^ However, semaglutide 2.4 mg weekly compared to placebo has not proven to improve fibrosis or resolve MASH in individuals with cirrhosis.^311^ Retrospective case-control studies in T2DM (not specific for cirrhosis) have reported a lower risk of LREs in individuals treated with SGLT2 inhibitors.^142^^,^^312^

#### Dyslipidaemia

The safety of statins and the absence of an increased risk of hepatotoxicity are well-established, including in individuals with compensated cirrhosis.^153^ Retrospective observational studies indicate a potential benefit of statins in preventing HCC and LREs in individuals with cirrhosis.^6^ However, in individuals with decompensated cirrhosis, high doses of statins should be avoided, as they are associated with an increased risk of severe adverse events.^153^^,^^308^^,^^313^^,^^314^

#### Anti-obesity drugs

There is currently no robust evidence supporting the use of anti-obesity pharmacotherapy for the treatment of MASH in individuals who have progressed to cirrhosis. While GLP-1RAs and other anti-obesity agents have demonstrated benefit in MASH and fibrosis regression in non-cirrhotic populations (see section on antidiabetic and anti-obesity therapies), their safety and efficacy in individuals with cirrhosis – especially decompensated cirrhosis – are not established, and these agents should, in the absence of sufficient evidence, not be used in cirrhosis at this point.^315^

### Specific MASH treatment

To date, for individuals with cirrhosis, no drugs have demonstrated benefit on MASH-specific clinical trial endpoints in phase III.^316^ Semaglutide 2.4 mg once weekly for 1 year did not induce histological fibrosis regression, nor improvement in collagen content or liver stiffness, suggesting an absence of efficacy as mentioned before.^311^ On the other hand, in line with the demonstrated benefit of weight loss on HVPG in a small cohort study of mixed aetiology,^256^ a potential long-term benefit in terms of portal hypertension-related complications cannot be excluded. Given this consideration and the demonstrated safety, it can hence be used as an anti-obesity treatment in individuals with MASH-related cirrhosis, carefully considering the potential issue of aggravating sarcopenia. However, as stated, in the absence of sufficient evidence, anti-obesity therapy should be avoided in individuals with cirrhosis if there is no scientifically proven benefit.

Several compounds with different modes of action are currently being tested in MASH-related cirrhosis and may offer future treatment opportunities.^317^^,^^318^

## Conclusions

**Fig. 10:**
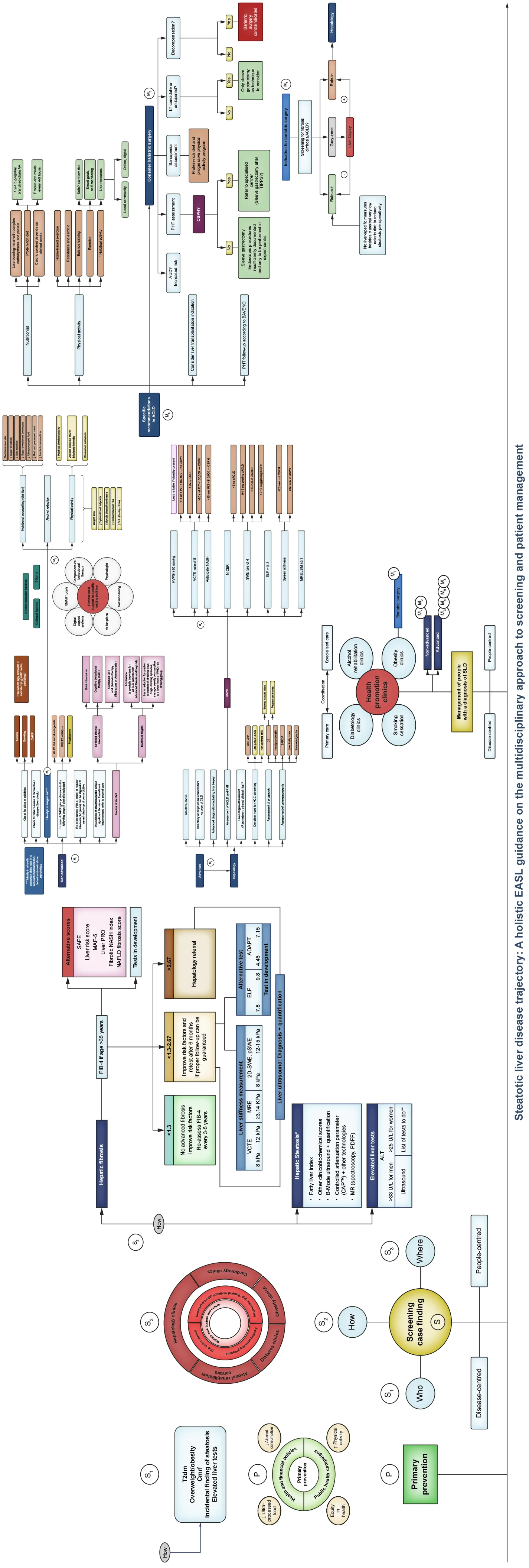
**Detailed SLD trajectory, integrating all subparts from the summary provided in**Fig. 1. Primary prevention (P); Screening (S): Who (S1), How (S2), Where (S3); Management (M), Management of people living with SLD with non-advanced disease (M1), Lifestyle management (M2), Management of people living with SLD with advanced disease (M3), Management of people living with SLD with CSPH. (M4), Management of people living with SLD and ACLD (M5), Management of bariatric surgery in people living with SLD (M6-7). 2D-SWE, two-dimensional shear wave elastography; AA, amino acid; ACLD, advanced chronic liver disease; AFP, alpha-fetoprotein; ALT, alanine aminotransferase; AUD, alcohol use disorder; cACLD, compensated advanced chronic liver disease; CAP, controlled attenuation parameter; CBT, cognitive behavioural therapy; CEUS, contrast-enhanced ultrasound; CLD, chronic liver disease; CMRF, cardiometabolic risk factor(s); CSPH, clinically significant portal hypertension; ELF, enhanced liver fibrosis (test); FIB-4, fibrosis-4 index; GLP1 RA, glucagon-like peptide-1 receptor agonist; HCC, hepatocellular carcinoma; HVPG, hepatic venous pressure gradient; LSM, liver stiffness measurement; LT, liver transplantation; MAF-5, metabolic dysfunction-associated fibrosis-5 (score); MRE, magnetic resonance elastography; NAFLD, non-alcoholic fatty liver disease; NASH, non-alcoholic steatohepatitis; PDFF, proton density fat fraction; PHT, portal hypertension; PLT, platelets; pSWE, point shear wave elastography; QoL, quality of life; SGLT2, sodium-glucose cotransporter-2 (inhibitor); SLD, steatotic liver disease; T2DM, type 2 diabetes mellitus; TIPS, transjugular intrahepatic portosystemic shunt; US, ultrasound; VCTE, vibration-controlled transient elastography.

The goal of this guidance is to provide, to all HCPs in their respective settings, recommendations on all aspects of care for people at risk of developing, presenting with, or experiencing complications from SLD. In the current guidance, the full spectrum of individuals living with SLD is discussed from screening, prevention and management of liver disease in the primary care setting up to management of decompensated SLD-related cirrhosis in tertiary care settings. Here, we highlight different aspects of healthcare in which it is evident that SLD requires a multidisciplinary approach, including, but not limited to, general practitioners, addiction unit professionals, dietitians, physiotherapists, metabolic health specialists and hepatologists. The fully executed trajectory is depicted in Fig. 10, compiling previous figures and detailing the summary provided in Fig. 1. The recommendations in this guidance are focused on “must-haves” and “nice-to-haves”, making it applicable to different healthcare systems and resource settings across the globe. Importantly, socio-economic inequities in SLD persist across all levels of care, while those most affected remain insufficiently reached despite WHO’s call for more precise, equity-focused action. This gap should be addressed, which requires a fundamental shift from disease-centred healthcare toward holistic, people-centred care embedded within truly integrated health systems.

## Abbreviations

ALD, alcohol-related liver disease; ALT, alanine aminotransferase; ASCVD, atherosclerotic CVD; AUD, alcohol use disorder; AUDIT, Alcohol Use Disorders Identification Test; AUDIT-C, Alcohol Use Disorders Identification Test-Consumption; BMI, body mass index; cACLD, compensated advanced chronic liver disease; CHB, chronic hepatitis B; CSPH, clinically significant portal hypertension; CVD, cardiovascular disease; ELF, Enhanced Liver Fibrosis; FIB-4, fibrosis-4; GLP-1 RA, glucagon-like peptide-1 receptor agonist; HCC, hepatocellular carcinoma; HCP, healthcare professional; HPC, health promotion centre; LSM, liver stiffness measurement; MASLD, metabolic dysfunction-associated steatotic liver disease; MASH, metabolic dysfunction-associated steatohepatitis; MetALD, MASLD with increased alcohol intake; MetS, metabolic syndrome; MHS, metabolic health specialist; MRE, magnetic resonance elastography; NCDs, non-communicable diseases; NITs, non-invasive tests; NPV, negative predictive value; PEth, phosphatidylethanol; PPV, positive predictive value; SLD, steatotic liver disease; SWE, shear wave elastography; SGLT2, sodium-glucose cotransporter 2; T2DM, type 2 diabetes mellitus; TIPS, transjugular intrahepatic portosystemic shunt; fVCTE, vibration-controlled transient elastography.

## Authors’ contributions

Working groups are outlined in the supplementary file.

## Financial support

No funding was received for the development of this guidance. The in-person meeting in the context of the EASL SLD Summit benefitted from the overall organisation of the summit, without any involvement in the process before or after the meeting and without any impact on the content of this guidance.

## Conflicts of interest

WPB has served as speaker for Novo Nordisk, Boehringer Ingelheim, Eli Lilly and participated in advisory boards of Novo Nordisk; participates in trials of Inventiva Pharma, 89BIO, Boehringer Ingelheim, all COI are outside the submitted work. LV has acted as consultant for Abbvie, Bayer, Eisai, Inventiva, Siemens and has received grants/fees from Abbvie, Bayer, Gilead, Merk, Norgine, GE HealthCare, PanNASH initiative. MT speaker's fee from Echosens, Madrigal, Takeda, and Novo Nordisk. Advisory fee from Boehringer Ingelheim, Astra Zeneca, Novo Nordisk and GSK. Investigator-initiated research grant from GSK. Co-founder of Evido. JMS declares consultant honorary from Akero, Alentis, Alexion, Altimmune, Astra Zeneca, 89Bio, Bionorica, Boehringer Ingelheim, Boston Pharmaceuticals, Gilead Sciences, GSK, HistoIndex, Ipsen, Inventiva Pharma, Madrigal Pharmaceuticals, Kríya Therapeutics, Eli Lilly, MSD Sharp & Dohme GmbH, Novartis, Novo Nordisk, Pfizer, Roche, Sanofi, Siemens Healthineers; speaker honorarium from AbbVie, Boehringer Ingelheim, Gilead Sciences, Ipsen, Lilly, Madrigal Pharmaceuticals, MSD, Novo Nordisk. Stockholder options: Hepta Bio. AF has participated in advisory board organized by Reckitt. SB has been lecturer for following Swiss Universities of Applies Sciences: Zürcher Hochschule für Angewandte Wissenschaften, Scuola universitaria professionale della Svizzera italiana, and Hochschule Luzern. EB has served as speaker for Boehringer Ingelheim, Eli Lilly, Madrigal, MSD and Novo Nordisk and participated in advisory boards for Boehringer Ingelheim, Eli Lilly, Madrigal, MSD and Novo Nordisk, all outside the submitted work. CL consults for and is on the speakers’ bureau for Boehringer Ingelheim, Echosens, Madrigal, Gilead, and Novo Nordisk. He consults for Boston Pharmaceuticals, GSK, MSD, Novo Nordisk, Sagimet, Siemens, and Pfizer. He is on the speakers’ bureau for AstraZeneca and Inventiva. CC received consultant fees from Gilead, NovoNordisk, AstraZeneca, Lilly, E-scopics, MSD, Bayer, Corcept, Echosens, Madrigal, Boerhinger Ingelhaim, grant support from Gilead; NovoNordisk, Echosens. AC has served as a speaker for GSK, MSD, Gilead Sciences, and Eva Pharma, and participated in advisory boards for GSK, MSD, Gilead Sciences, and Eva Pharma, all outside the submitted work. Research support to institution; Eva Pharma and Roche. RDB holds a senior clinical investigator fellowship from the Research Foundation Flanders (FWO) (1843824N). Her institution has received consulting and lecture fees from Orphalan, Mirum Pharma and Ipsen and a grant from Mirum Pharma. FN has no conflict of interest. GF reports research grants paid to the Institution from Spanish Institute of Health ISCIII. Payment of honoraria from Eli Lilly, Novo Nordisk, Regeneron, Astra Zeneca, Boehringer Ingelheim and Marabou Foundation as speaker and/or member of advisory boards, and payment of honoraria as member of the OPEN Spain Initiative. Co-chair of the Scientific Advisory Board of the European Society for the Study of Obesity - unpaid position. ZG has no conflict of interest. AG is consultant for Boehringer Ingelheim, Eli Lilly and Company, Merck Sharp & Dohme, Novo Nordisk, Regeneron Pharmaceuticals, Pfizer lectures; received lecture fees from Boehringer Ingelheim, Merck Sharp & Dohme, Novo Nordisk, Madrigal, Eli Lilly, Catalyst Medical Education, Translational Medical Academy Foundation. AG holds a senior clinical investigator fellowship from the Research Foundation Flanders (FWO) (1805718N). LH has no conflict of interest. KH has no conflict of interest. AH received research grants from Gilead and Novo Nordisk, acted as consultant for Gilead, Echosens, Novo Nordisk, Norgine, Julius Clinical, Inventiva and Boehringer Ingelheim, (co)leads the LEGEND trial with Inventiva and the SYNCH trial with Caelus Health and the Akkermansia Company and participates in trials of 89BIO, Boehringer Ingelheim, Novo Nordisk, and Inventiva. CL receives consultant fees for Dr Falk Pharma, Regeneron, Akero, Bio89, Boeringer Ingelheim, Inventiva and IQVIA Biotech LLC. JVL acknowledges grants to ISGlobal from AbbVie, Boehringer Ingelheim, Echosens, Gilead Sciences, Madrigal Pharmaceuticals, MSD, Novo Nordisk, Pfizer and Roche Diagnostics, consulting fees from Echosens, GSK, Novo Nordisk, Pfizer and Prosciento and payment or honoraria for lectures from AbbVie, Echosens, Gilead Sciences, Janssen, Moderna, MSD, Novo Nordisk and Pfizer. He also acknowledges institutional support to ISGlobal from grant CEX2023-0001290-S, funded by MCIN/AEI/10.13039/501100011033, and the Generalitat de Catalunya, through the CERCA Programme. JM has no conflict of interest. MM declares consultant honorary from Boehringer Ingelheim and Ipsen; Speaker honorarium from AbbVie, AstraZeneca, Daiichi Sankyo, Gilead Sciences, GSK, Ipsen and NovoNordisk; Travel support: Abbvie, Boehringer Ingelheim, Gilead Sciences, Ipsen, Mirum and NovoNordisk. VM reports engagement on advisory boards and/or has received honoraria for educational events from Boehringer Ingelheim, Roche, Novo Nordisk, Eli Lilly, Boston Scientific, and consultancy from ECPO, all unrelated to this work. VR has done scientific consulting for Novo Nordisk, Boehringer Ingelheim, Glaxo-Smith-Kline, 89bio, Sagimet Biosciences, Akero Therapeutics, Madrigal Pharmaceuticals, Hanmi; and has received institutional grant support from MSD. MR received lecture fees or served on advisory boards for AstraZeneca, Boehringer Ingelheim, Echosens, Eli Lilly, Madrigal, Merck-MSD, Novo Nordisk, Sanofi-Aventis, Synlab and Target RWE and performed investigator-initiated research with support from Boehringer Ingelheim, Novo Nordisk and Sanofi-Aventis to the German Diabetes Center (DDZ). MR received lecture fees or served on advisory boards for AstraZeneca, Boehringer Ingelheim, Echosens, Eli Lilly, Madrigal, Merck-MSD, Novo Nordisk, Synlab and Target RWE and performed investigator-initiated research with support from Boehringer Ingelheim, Novo Nordisk and Sanofi to the German Diabetes Center (DDZ). AJS has owner ship interests in Tiziana, Durect, NorthSea, Rivus, Inversago. He has consulted for Intercept, Merck, Novo Nordisk, Boehringer Ingelhiem, Akero, 89Bio, Boston Pharma, Altimuune, Regeneron, Eli Lilly, Genentech, Ipsen, Inventiva, Hanmi, LG Chem, Takeda, Astra Zeneca, Surrozen, Pliant, Avant Sante, Myovant, Corcept, Zydus, Madrigal, Path AI, Histoindex, Salix, Target RWE, Glaxo Smith Kline, Amgen. His institution has received grants from Merck, Echosens, Hepatoscope, Hanmi, Madrigal, Novo Nordisk, Boehringer Ingelhiem, Akero, 89 Bio, Inventiva, Takeda, Histoindex. He has royalties from Elsevier and Wolter Kluwers. CWS has received honoraria for Lectures on MASLD from Novo Nordisk and Sanofi. HS received lecture fees from Abbvie, Echosens, General Electric, Gilead Sciences and MSD, and received support from e-Scopics for performing investigator-initiated research. DT is consultant for ICON plc and Inventiva Pharma. ET acknowledges advisory boards for Boehringer, Siemens Healthineers, MSD, and NovoNordisk and speaker fees for Astra Zeneca, Boehringer, Echosens, Novo Nordisk, and AbbVie. JW has no conflict of interest. FZ consulting fees from Applied Therapeutics, Bayer, Bristol Myers Squibb, Boehringer Ingelheim, Boston Scientific, Cardior Pharmaceuticals, Cereno Scientific, Cellprothera, CVRx, Merck, Novartis, Pfizer and Servier and is the co-founder of cardiorenal and the founder of CVCT. SZS reports being a researcher at the Global NASH/MASH Council; being a public health counsellor for the European Association for the Study of the Liver; and research grants from the Israeli Ministry of Health and Chief Scientist's Office. AK has served as speaker for Novo Nordisk, Norgine and participated in advisory boards for Boehringer Ingelheim, GSK, W4Cure, Madrigal and Novo Nordisk, all outside the submitted work. Research support to institution; Astra, Siemens, Nordic Bioscience, GSK, Echosense. Board member and co-founder Evido. SF holds a senior clinical investigator fellowship from the Research Foundation Flanders (FWO) (1802154N). His institution has received grants from Astellas, Falk Pharma, Genfit, Gilead Sciences, GlympsBio, Janssens Pharmaceutica, Inventiva, Merck Sharp & Dome, Pfizer, Roche. He has acted as consultant for Abbvie, Actelion, Aelin Therapeutics, AgomAb, Aligos Therapeutics, Allergan, Alnylam, Astellas, Astra Zeneca, Bayer, Boehringer Ingelheim, Bristoll-Meyers Squibb, Byondis, CSL Behring, Coherus, Echosens, Dr. Falk Pharma, Eisai, Enyo, Galapagos, Galmed, Genetech, Genfit, Genflow Biosciences, Gilead Sciences, Intercept, Inventiva, Janssens Pharmaceutica, PRO.MED.CS Praha, Julius Clinical, Madrigal, Medimmune, Merck Sharp & Dome, Mursla Bio, NGM Bio, Novartis, Novo Nordisk, Promethera, Roche, Siemens Healthineers, V4Cure, Weatherden. SF has been lecturer for Abbvie, Allergan, Bayer, Eisai, Genfit, Gilead Sciences, Janssens Cilag, Intercept, Inventiva, Merck Sharp & Dome, Novo Nordisk, Promethera, Siemens.

Please refer to the accompanying ICMJE disclosure forms for further details.
