# Corrigendum to ‘Steatotic liver disease trajectory: A holistic EASL guidance on the multidisciplinary approach to screening and patient management’ [JHEP Reports (2026) 8; 101798; https://doi.org/10.1016/j.jhepr.2026.101798]

**DOI:** 10.1016/j.jhepr.2026.102061

**Published:** 2026-10-30

**Authors:** Willem Pieter Brouwer, Luisa Vonghia, Maja Thiele, Jörn Schattenberg, Foteini Anastasiou, Sonja Beckmann, Elisabetta Bugianesi, Laurent Castera, Cyrielle Caussy, Ahmed Cordie, Ruth De Bruyne, Núria Fabrellas, Gema Frühbeck, Zouhir Gadi, Amalia Gastaldelli, Anja Geerts, Laura Haigh, Kate Hallsworth, Adriaan Holleboom, Carolin Lackner, Jeffrey V. Lazarus, Juan Mendive, Maurice Michel, Vicki Mooney, Vlad Ratziu, Michael Roden, Arun J. Sanyal, Wendy C. Spearman, Horia Stefanescu, Dina Tiniakos, Emmanuel Tsochatzis, José Willemse, Faiez Zannad, Shira Zelber-Sagi, Aleksander Krag, Sven Francque

**Affiliations:** 1 Erasmus MC University Medical Center, Rotterdam, The Netherlands; 2 Department of Gastroenterology and Hepatology, Antwerp University Hospital, Antwerp, Belgium; 3 InflaMed Centre of Excellence, Laboratory of Experimental Medicine and Paediatrics, University of Antwerp, Antwerp, Belgium; 4 Center for Liver Research, Odense University Hospital and University of Southern Denmark, Odense, Denmark; 5 Department of Internal Medicine II, Saarland University Medical Center, Homburg, Germany; 6 4rth TOMY –Municipality & Academic Practice of Heraklion Crete, Greece, European Society for Primary Care Gastroenterology, Greece; 7 Center for Clinical Nursing Science & Department of Gastroenterology and Hepatology; University Hospital Zurich, Switzerland; 8 Department of Medical Sciences, University of Torino, Italy; 9 Université Paris-Cité, Hôpital Beaujon, Service d'hépatologie, Assistance Publique-Hôpitaux de Paris, Clichy, France; 10 INSERM Unité Mixte de Recherche 1149, Clichy, France; 11 Universite Claude Bernard Lyon 1, CarMen Laboratory, INSERM, INRAE, Pierre-Bénite, France; 12 Hospices Civils de Lyon, Département Endocrinologie, Diabète et Nutrition, Hôpital Lyon Sud, Pierre-Bénite, France; 13 Cairo University Hospitals HIV Clinic, Department of Endemic Medicine, Faculty of medicine, Cairo University, Egypt; 14 Department of Pediatric Gastroenterology and Hepatology, Ghent University Hospital; Department of Internal Medicine and Pediatrics, Ghent University, Ghent, Belgium; 15 Liver Unit, Hospital Clínic of Barcelona, Barcelona, Catalunya, Spain; 16 Institut d'Investigacions Biomèdiques August Pi i Sunyer (IDIBAPS), Barcelona, Catalunya, Spain; 17 Faculty of Nursing, University of Barcelona, Barcelona, Spain; 18 Department of Endocrinology & Nutrition, Clínica Universidad de Navarra, IdiSNA, CIBEROBN, Instituto de Salud Carlos III; Institute for Nutrition & Health, University of Navarra, Pamplona, Spain; 19 Research Council (CNR), Institute of Clinical Physiology (IFC), Pisa, Italy; 20 Hepatology Research Unit, Department of Internal Medicine and Pediatrics, Ghent University, Ghent, Belgium; 21 Liver Research Center Ghent, Ghent University Hospital, Ghent, Belgium; 22 Newcastle NIHR Biomedical Research Centre, Newcastle upon Tyne Hospitals, NHS Foundation Trust, Newcastle upon Tyne, UK; 23 Translational and Clinical Research Institute, Faculty of Medical Sciences, Newcastle University, Newcastle upon Tyne, UK; 24 NIHR Newcastle Biomedical Research Centre, Newcastle upon Tyne Hospitals; NHS Foundation Trust, Newcastle upon Tyne, UK; 25 Department of Vascular Medicine, Amsterdam UMC, Amsterdam, The Netherlands; 26 Amsterdam Gastroenterology Endocrinology and Metabolism Institute, Amsterdam UMC, Amsterdam, The Netherlands; 27 Institute of Pathology, Medical University of Graz, Austria; 28 City University of New York Graduate School of Public Health and Health Policy (CUNY SPH), New York, NY, USA; 29 Barcelona Institute for Global Health (ISGlobal), Barcelona, Spain; 30 Faculty of Medicine and Health Sciences, University of Barcelona (UB), Barcelona, Spain; 31 La Mina Primary Care Academic Centre, Catalan Health Institute and Institute for Primary Health Care Research IDIAP Jordi Gol, Barcelona, Spain; 32 European Coalition for People living with Obesity, Dublin, Ireland; 33 Institute for Cardiometabolism and Nutrition, Sorbonne Université and Hospital Pitié Salpêtrière, Paris, France; 34 Department of Endocrinology and Diabetology, Medical Faculty and University Hospital Düsseldorf, Heinrich-Heine-University Düsseldorf, Düsseldorf, Germany; 35 Institute for Clinical Diabetology, German Diabetes Center, Leibniz Center for Diabetes Research, Heinrich Heine University, Düsseldorf, Germany; 36 German Center for Diabetes Research (DZD e.V.), Partner Düsseldorf, Germany; 37 Stravitz-Sanyal Institute for Liver Disease and Metabolic Health, Div. of Gastroenterology, Hepatology and Nutrition, VCU School of Medicine, Richmond, VA, USA; 38 Division of Hepatology, Department of Medicine, Faculty of Health Sciences, University of Cape Town, Cape Town, South Africa; 39 Liver Unit, Regional Institute of Gastroenterology and Hepatology, Cluj-Napoca, Romania; 40 Department of Pathology, Aretaieion Hospital, Medical School, National and Kapodistrian University of Athens, Athens, Greece; 41 UCL Institute for Liver and Digestive Health, Royal Free Hospital and UCL, London, UK; 42 European Reference Network of Hepatological Diseases (ERN-RARE LIVER), Hamburg, Germany; 43 Dutch Liver Patients Association, Hoogland, The Netherlands; 44 Centre d'Investigation Clinique Plurithémathique Pierre Drouin and Département de Cardiologie, Institut Lorrain du Coeur et des Vaisseaux, CHU de Nancy, Nancy, France; 45 The Global NASH/MASH Council, Washington, DC, USA; 46 School of Public Health, University of Haifa, Haifa, Israel; 47 Fibrosis, Fatty Liver and Steatohepatitis Research Center Odense (FLASH), Department of Gastroenterology and Hepatology, Odense University Hospital, Odense, Denmark; 48 Department of Clinical Research, Faculty of Health Sciences, University of Southern Denmark, Odense, Denmark

It has come to our attention that an author's surname was incorrectly spelled in the original publication. The author's name was published as “Tsochatsis” and should have read “Tsochatzis.” This has been corrected in the online version of the article. The authors apologise for any inconvenience caused.

