# Supplementary material for: Steatotic liver disease trajectory: A holistic EASL guidance on the multidisciplinary approach to screening and patient management
Source: JHEP Rep. 2026 Feb 25;8(9):101798. doi: 10.1016/j.jhepr.2026.101798 (PMC13521106; doi:10.1016/j.jhepr.2026.101798)
Supplement: Supplementary file 1 [file mmc1.pdf]

Steatotic liver disease trajectory: A holistic EASL guidance on the multidisciplinary approach to screening and patient management

Willem Pieter Brouwer, Luisa Vonghia, Maja Thiele, Jörn Schattenberg, Foteini Anastasiou, Sonja Beckmann, Elisabetta Bugianesi, Laurent Castera, Cyrielle Caussy, Ahmed Cordie, Ruth De Bruyne, Núria Fabrellas, Gema Frühbeck, Zouhir Gadi, Amalia Gastaldelli, Anja Geerts, Laura Haigh, Kate Hallsworth, Adriaan Holleboom, Carolin Lackner, Jeffrey V. Lazarus, Juan Mendive, Maurice Michel, Vicki Mooney, Vlad Ratziu, Michael Roden, Arun J. Sanyal, Wendy C. Spearman, Horia Stefanescu, Dina Tiniakos, Emmanuel Tsochatsis, José Willemse, Faiez Zannad, Shira Zelber-Sagi, Aleksander Krag, Sven Francque

Table of content

Table S1……………………………………………………………………………………2

Table S1.
Working groups composition

**Senior authors**	Francque Sven
	Krag Aleksander
**Secretaries**	Brouwer Willem Pieter
	Vonghia Luisa
**Working group 1**	**Screening and case-finding strategies**
Maja Thiele	teamleader
Brouwer Willem Pieter	
Cordie Ahmed	
De Bruyne Ruth	
Fabrellas Núria	
Francque Sven	
Frühbeck Gema	
Holleboom Adriaan	
Krag Aleksander	
Lazarus Jeffrey V.	
Mendive Juan	
Mooney Vicki	
Sanyal Arun J.	
Schattenberg Jörn	
Stefanescu Horia	
Tiniakos Dina	
Tsochatsis Emmanuel	
Vonghia Luisa	
Zannad Faiez	
**Working group 2**	**Management at different stages of disease identification: how and who**
Schattenberg Jörn	teamleader
Anastasiou Foteini	
Beckmann Sonja	
Brouwer Willem Pieter	
Bugianesi Elisabetta	
Castera Laurent	
Caussy Cyrielle	
Cordie Ahmed	
De Bruyne Ruth	
Fabrellas Nuria	
Francque Sven	
Gastaldelli Amalia	
Haigh Laura	
Hallsworth Kate	
Krag Aleksander	

Lackner Carolin	
Michel Maurice	
Mooney Vicki	
Ratziu Vlad	
Roden Michael	
Sanyal Arun J.	
Spearman C. Wendy	
Stefanescu Horia	
Tiniakos Dina	
Vonghia Luisa	
Willemse José	
Zannad Faiez	
Zelber-Sagi Shira	
**Working group 3**	**Cirrhosis and compensated advanced chronic liver disease**
Francque Sven	teamleader
Anastasiou Foteini	
Brouwer Willem Pieter	
Castera Laurent	
Caussy Cyrielle	
Cordie Ahmed	
Fabrellas Núria	
Gadi Zouhir	
Geerts Anja	
Haigh Laura	
Hallsworth Kate	
Krag Aleksander	
Lackner Carolin	
Roden Michael	
Ratziu Vlad	
Roden Michael	
Sanyal Arun J.	
Spearman C. Wendy	
Stefanescu Horia	
Tiniakos Dina	
Tsochatsis Emmanuel	
Vonghia Luisa	
Willemse José	
Zelber-Sagi Shira	
